# Assessment of heated herbal products’ tobacco harm reduction potential: pre‐clinical and clinical studies

**DOI:** 10.3389/ftox.2025.1589480

**Published:** 2025-08-11

**Authors:** Alvaro-Flavio Marinas-Lacasta, Ian M. Fearon, Matthew Stevenson, Tasnim Abusalem, Fiona Chapman, Edgar Trelles Sticken, Roman Wieczorek, Sarah Jean Pour, Ole Dethloff, Ourania Komini, Mike Brown, Liam Simms, Thomas Nahde

**Affiliations:** ^1^ Imperial Brands PLC, Bristol, United Kingdom; ^2^ WhatIF? Consulting Ltd., Harwell, United Kingdom; ^3^ Reemtsma Cigarettenfabriken GmbH, An Imperial Brands PLC company, Hamburg, Germany

**Keywords:** cigarette smoking, tobacco harm reduction, heated herbal product, analytical chemistry, toxicology, abuse liability

## Abstract

Data from pre-clinical and clinical studies form part of an integrated assessment of the tobacco harm reduction (THR) potential of novel products that may act as cigarette alternatives for adult smokers. We report data from pre-clinical (emissions chemistry and *in vitro* toxicology) and clinical (nicotine pharmacokinetics and subjective effects) studies conducted with the iSENZIA™ heated herbal system (HHS; PULZE™ 2.0 device with iSENZIA™ sticks), which utilizes electronic heating of a tea-based substrate to generate an inhalable nicotine-containing aerosol. The aerosols from the iSENZIA™ HHS contained significantly lower levels, by up to 99.8%, of the nine World Health Organization Study Group on Tobacco Product Regulation (WHO TobReg) analytes compared with 1R6F reference cigarette smoke and elicited significantly lower *in vitro* cytotoxicity, genotoxicity, and mutagenicity responses. The clinical study demonstrated that the iSENZIA™ HHS delivers satisfactory levels of nicotine to users and has lower abuse liability than cigarettes. Overall, our data suggest that iSENZIA™ has the potential to offer substantially reduced toxicant exposure, as well as a reduction in toxicity, compared to cigarettes, while delivering satisfactory levels of nicotine. These findings support the THR potential of the iSENZIA™ HHS as a reduced-risk, acceptable alternative product for adult smokers.

## Introduction

Smoking is a cause of serious diseases in smokers, including lung cancer, cardiovascular disease, and emphysema. Cigarette smoking is reported as the world’s leading cause of preventable deaths (International Agency fo r Research on Cancer, 2007; US Department of Health and Human Services, 2014; World Health Organization, 2011; World Health Organization, 2023), responsible for more than 7 million deaths per year globally (World Health Organization, 2023). The greatest risk of smoking-related diseases comes from burning tobacco and inhaling smoke containing approximately 7,000 chemicals. One component of cigarette smoke is nicotine. While nicotine is an addictive chemical and not risk-free, it is not thought to be directly responsible for the harmful effects of cigarette smoking (Abrams et al., 2018; Gottlieb and Zeller, 2017; Royal College of Physicians, 2016). A number of chemicals identified in cigarette smoke (Perfetti and Rodgman, 2011; Toll et al., 2024) have been termed harmful and potentially harmful constituents (HPHCs) and are linked to cardiovascular and respiratory diseases, lung cancer, and reproductive/developmental toxicity (Food and Drug Administration, 2012). The best possible action for smokers to reduce or eliminate exposure to HPHCs and other toxicants, and therefore to reduce disease risk, is to stop smoking (US Department of Health and Human Services, 2014). However, although a large proportion of smokers report intending to take this course of action or begin a quit attempt each year, only a small fraction of smokers manage to successfully quit smoking (Babb et al., 2017; Centers for Disease Control and Prevention, 2021; Centers for Disease Control and Prevention, 2022; Pierce, 2022).

For those smokers who are unwilling or uninterested in quitting, a tobacco harm reduction (THR) approach to reducing the harms associated with smoking has been proposed as beneficial. The THR concept of reducing exposure to harmful toxicants as a means of reducing morbidity and mortality was first highlighted by the US Institute of Medicine (IoM) in 2001 (Institute of Medicine, 2001; Stratton et al., 2001) in their ‘Clearing the Smoke’ report. In this report, the IoM defined THR as “*minimizing harms and decreasing total morbidity and mortality, without completely eliminating tobacco and nicotine use*,” and suggested that while a significant proportion of individuals will continue to use tobacco, THR can be achieved by decreasing the risk associated with tobacco use, by decreasing the users’ consumption, or by decreasing the prevalence of use (Institute of Medicine, 2001). This gives rise to the concept that both the individual- and population-level health impacts of smoking can be reduced by novel nicotine and tobacco products that deliver nicotine without exposing the user to the HPHCs and other toxicants responsible for smoking-related diseases. In recent years, there has been increased support for a toxicant exposure reduction approach to THR, and a number of global public health authorities now advocate for this approach (Royal College of Physicians, 2016; Health Canada, 2022; New Zealand Ministry of Health, 2020; Office for Health Improvement and Disparities, 2022), while other global health authorities have been called upon to make THR a central strategy in promoting public health (Beaglehole and Bonita, 2024; Warner, 2019; Yach, 2022).

One alternative form of nicotine-containing product for adult smokers who would otherwise continue to smoke is heated tobacco products (HTPs), which electrically heat a tobacco-based substrate in a controlled manner to produce a nicotine-containing aerosol (Eaton et al., 2018; Jaccard et al., 2017; Schaller J. P. et al., 2016; Cordery et al., 2024; Smith et al., 2016). Because this heating occurs at temperatures that are significantly lower than those that cause combustion in cigarettes, the levels of HPHCs and other toxicants are significantly lower in HTP aerosols than the levels found in cigarette smoke (Chapman et al., 2023a; Forster et al., 2018; Malt et al., 2022; Poynton et al., 2017; Szparaga et al., 2021). This results in reduced toxicant exposure among smokers who exclusively switch to using HTPs (Gale et al., 2021a; Gale et al., 2021b; Gale et al., 2019; Gale et al., 2022; Haziza et al., 2020a; Haziza et al., 2016; Haziza et al., 2017; Lüdicke et al., 2018a). Furthermore, data from a number of pre-clinical and clinical studies conducted to date support the potentially reduced risk profile and THR potential of HTPs (Cordery et al., 2024). An emerging category of heated nicotine delivery products for adult smokers who would otherwise continue to smoke is heated herbal products (HHPs). Instead of heating tobacco, HHPs electronically heat a non-tobacco, nicotine-containing substrate, at temperatures lower than those which allow combustion, to produce an aerosol containing nicotine. A tobacco-derived nicotine salt, compliant with pharmaceutical-grade quality standards, is added to the substrate of the HHP. To date, however, unlike HTPs, there are no published studies assessing HHPs. In this article, we describe the findings from a suite of pre-clinical (emissions chemistry and *in vitro* toxicology) studies, and a clinical study assessing nicotine pharmacokinetics and subjective effects, conducted with a novel heated herbal system (HHS), iSENZIA™, which is comprised of the PULZE™ 2.0 device and iSENZIA™ sticks. The data arising from these multidisciplinary studies provide the first evidence of the THR potential of the iSENZIA™ HHS and contribute to the growing body of evidence for this novel product category.

## Results

### Pre-clinical studies

#### Emissions chemistry assessment

**FIGURE 1:**
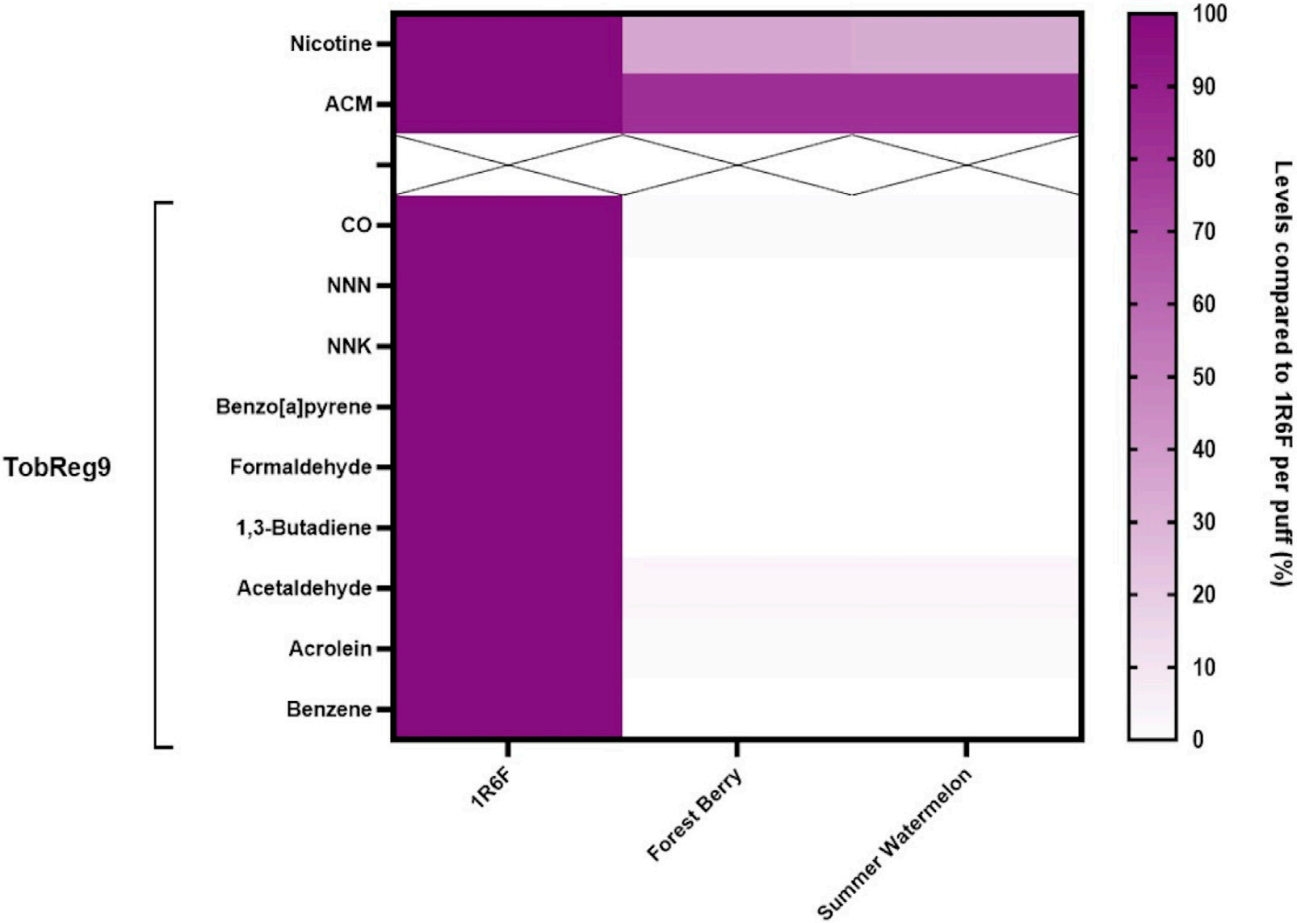
Heatmap of analytes in iSENZIA™ HHS *Forest Berry* and *Summer Watermelon* aerosol relative to 1R6F reference cigarette smoke. Data are presented as analyte levels in the iSENZIA™ *Forest Berry* or iSENZIA™ *Summer Watermelon* aerosol. Analyte levels are presented on a per-puff basis normalized to analyte levels in 1R6F reference cigarette smoke. 1R6F reference cigarettes were smoked on a rotary smoking machine according to the ISO 20778:2018 smoking regimen (55 mL puff volume, 2-s puff duration, 30-s puff interval, bell-shaped puff profile, filter vents blocked). The iSENZIA™ HHS was used on a linear smoking machine according to a modified version of ISO 20778:2018 (55 mL puff volume, 2-s puff duration, 30-s puff interval, bell-shaped puff profile, with no requirement for filter vent blocking). Abbreviations: ACM, aerosol collected mass; CO, carbon monoxide; HHS, heated herbal system; NNK, 4-(methylnitrosamino)-1-(3-pyridyl)-1-butanone; NNN, *N*-nitrosonornicotine; TPM, total particulate matter; WHO TobReg 9, World Health Organization Study Group on Tobacco Product Regulation proposal of toxicants mandated for lowering in cigarette smoke (Burns et al., 2008).

In the aerosols generated from the iSENZIA™ HHS with *Forest Berry* and *Summer Watermelon* sticks, six of the nine World Health Organization (WHO) Study Group on Tobacco Product Regulation (TobReg 9) analytes were below the limits of quantification (<LOQ; Supplementary Table S1): *N*′-nitrosonornicotine (NNN), 4-(methylnitrosamino)-1-(3-pyridyl)-1-butanone (NNK), benzo[a]pyrene (B[a]P), formaldehyde, 1,3-butadiene, and benzene. When comparing iSENZIA™ *Forest Berry* and iSENZIA™ *Summer Watermelon* aerosol analyte levels with those in 1R6F reference cigarette smoke, substantial reductions in all analytes were observed in the HHS aerosols on a per-puff and per-stick basis (Figure 1; Supplementary Table S1). For both varieties of iSENZIA™ HHS sticks assessed, percentage reductions in WHO TobReg 9 analyte levels for analytes that were >LOQ ranged from approximately 96.3% for acetaldehyde to approximately 98.8% for acrolein (Supplementary Table S1). The iSENZIA™ HHS aerosol nicotine yield was also substantially lower than that of 1R6F reference cigarette smoke by approximately 65% (*Forest Berry*) and 67% (*Summer Watermelon*). Aerosol collected mass (ACM) was lower for the iSENZIA™ HHS aerosols than total particulate matter (TPM) in 1R6F reference cigarette whole smoke by approximately 18% for both flavor variants (Supplementary Table S1).

### *In vitro* toxicology

#### Neutral red uptake cytotoxicity assay

**FIGURE 2:**
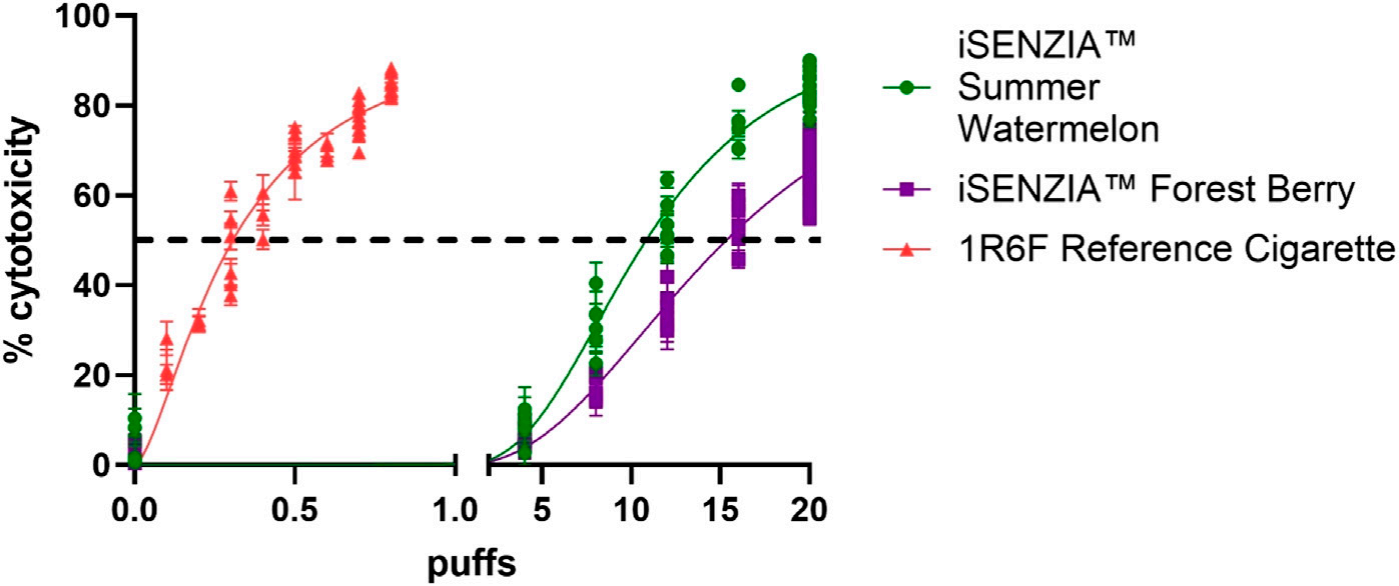
*Cytotoxicity* assessment of iSENZIA™ HHS *Forest Berry* and *Summer Watermelon* aerosol relative to 1R6F reference cigarette smoke. Percentage cytotoxicity induced in the neutral red uptake assay in Beas-2B cells following exposure to increasing puff numbers (log scale) of 1R6F reference cigarette whole smoke or iSENZIA™ HHS whole aerosol. Fifty percent cytotoxicity compared to the negative control (IC_50_) is marked with the black dotted line. Data shown are the mean +/− standard deviation with n = 3 (biological replicates). Generation of both cigarette smoke and iSENZIA™ HHS whole aerosol was performed in accordance with the ISO 20778:2018 smoking regimen. Abbreviation: HHS, heated herbal system.

Cytotoxicity assessments using the neutral red uptake (NRU) assay were conducted in Beas-2b cells following exposure to whole smoke/aerosol at the air–liquid interface (ALI). Cytotoxicity was observed (>IC_50_ achieved) following exposure to 1R6F reference cigarette whole smoke or iSENZIA™ HHS whole aerosols, and there were clear dose responses to all three test articles (Figure 2). The number of puffs required to induce IC_50_ was, on average, 50-fold and 35-fold higher for the iSENZIA™ HHS *Forest Berry* and *Summer Watermelon* aerosols, respectively, than for the 1R6F reference cigarette smoke (Figure 2).

#### Ames bacterial reverse mutation mutagenicity assay

**FIGURE 3:**
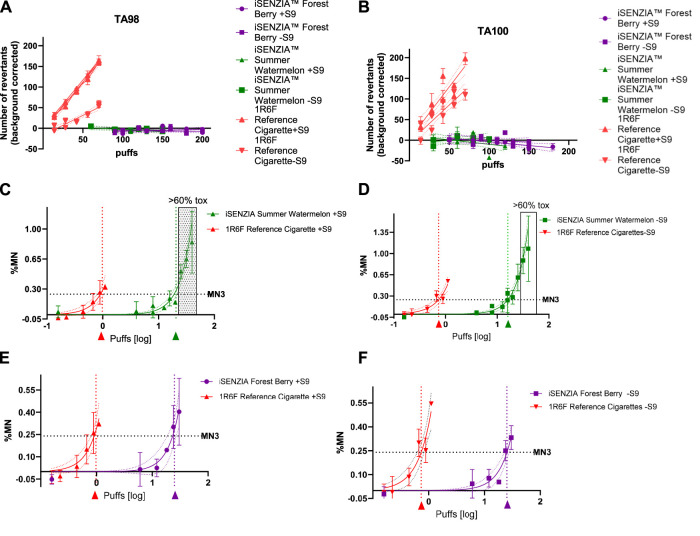
Mutagenicity and genotoxicity assessments of the iSENZIA™ HHS *Forest Berry* and *Summer Watermelon* aerosol relative to 1R6F reference cigarette smoke. Panels A and B show the average (background corrected) number of revertant colonies per plate in an Ames bacterial reverse mutation assay for TA98 **(A)** or TA100 **(B)** strains exposed to increasing numbers of puffs of 1R6F whole smoke or iSENZIA™ HHS whole aerosol, +/− S9 metabolic activation. n = 3 (technical replicates); error bars represent the standard deviation. Linear regression analysis was applied to the data (trendlines as indicated), and the slope was calculated. For the Ames assay, generation of fresh smoke from 1R6F reference cigarettes was performed according to the ISO3308:2012 smoking regimen, while for the iSENZIA™ HHS, ISO 20778:2018, without requiring vent blocking, was used to generate the aerosol. Background levels of spontaneous revertants for the Ames assay were: TA98 + S9: 33; TA98-S9: 22; TA100 + S9: 115; and TA100-S9: 100. **(C–F)** show the background subtracted micronucleus frequency in an *in vitro* micronucleus (IVM) assay in V79 cells following exposure to increasing puffs (log scale) of 1R6F whole smoke or HHS whole aerosol, +/− S9 metabolic activation. ECMN3 (number of puffs necessary to reach a three-fold increase over background micronucleus frequencies) analysis was carried out using nonlinear regression analysis (solid lines for each test article) to determine the puff-based concentration required to induce micronucleus frequencies three-fold over background levels for each test article. The dotted lines parallel to the y-axis intersect the ECMN3 values on the x-axis at the corresponding puff-based dose levels for 1R6F (red arrow) and the iSENZIA™ HHS variant (*Forest Berry* = purple arrow, *Summer Watermelon* = green arrow). The grey area indicates toxicity levels above 60%. n = 2 (biological replicates); error bars represent the standard deviation. For the IVM assay, generation of both cigarette smoke and HHS aerosol was performed in accordance with the ISO 20778:2018 smoking regimen. Historical background levels for the IVM assay were: V79-S9: 0.12%; V79 + S9: 0.14%. Abbreviation: HHS, heated herbal system.

Ames tests were conducted in two bacterial strains, TA98 and TA100, both with and without S9 metabolic activation. In either strain, both in the presence and absence of S9, 1R6F reference cigarette smoke induced significant increases in revertant colonies with increasing numbers of puffs (Figures 3A,B). This occurred over a lower range of exposure levels (puff numbers) than for either of the iSENZIA™ HHS variant aerosols, which did not induce significant increases in the number of revertant colonies in either bacterial strain, with and without S9, under the test conditions even at high puff numbers (Figures 3A,B). Overall, the assay was negative for both of the iSENZIA™ HHS variant aerosols.

### *In Vitro* micronucleus genotoxicity assay

The genotoxic activity of whole smoke/aerosol was assessed using *in vitro* micronucleus (IVM) assay in V79 cells, both with and without S9 metabolic activation. Significant responses were observed at substantially lower exposure levels (puff numbers) for the 1R6F reference cigarette smoke compared with either of the iSENZIA™ HHS aerosols, both with and without S9 activation (Figures 3C–F). Due to technical limitations, a short-term treatment in the absence of S9 was not applicable in parallel. As indicated by calculations of the number of puffs necessary to reach a three-fold increase over background micronucleus frequencies (ECMN3), 1R6F reference cigarette smoke exhibited substantially higher potency than either the iSENZIA™ HHS *Forest Berry* or *Summer Watermelon* aerosols, with ECMN3 values that were 24.8 fold and 21.4 fold higher, respectively, for the 1R6F reference cigarette smoke with S9 activation and 34.7 fold and 21.9 fold higher, respectively, for the 1R6F reference cigarette smoke without S9 activation (Figures 3C–F). Overall, the results for the assay were positive for both of the iSENZIA™ HHS variant aerosols and for the 1R6F reference cigarette.

### Clinical study

#### Subject demographics

Brief demographic details of the 25 subjects in the safety population are provided in Supplementary Table S2 for each randomization sequence and overall. Forty percent of subjects were male, and all subjects were White and not of Hispanic/Latino origin. No major differences in demographics were observed between the randomization sequence groups, although the mean age of the subjects and number of years smoking in one of the product sequence groups was higher than the other four groups (Supplementary Table S2). Of the 25 subjects, all smoked non-mentholated cigarettes as their usual brand.

#### Nicotine pharmacokinetics

**FIGURE 4:**
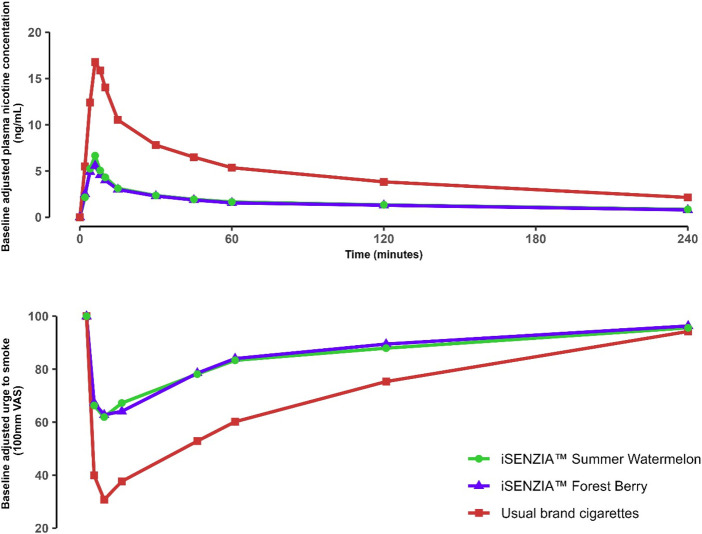
Baseline-adjusted plasma nicotine concentrations (upper) and urge to smoke (lower) over time following controlled product use in the outcomes population of the clinical study. N = 25 in each case. Data points are arithmetic means for each product at each time point. Error bars have been removed for clarity; for variability estimates, refer to Tables 2, 4. Abbreviation: VAS, visual analog scale.

Prior to the start of the controlled puffing sessions and following 12 h of abstinence from the use of any tobacco- or nicotine-containing products, the mean uncorrected plasma nicotine concentration was 1.06 ng/mL (standard deviation [SD] 0.756 ng/mL). During use of any of the study products in the controlled use sessions (puffs taken at 30-s intervals with puffs 3 s in duration), plasma nicotine levels rose rapidly (Figure 4). On average, during the controlled use session, subjects took 9.3 puffs on each of the iSENZIA™ stick variants and 10.3 puffs on their usual brand cigarettes (Supplementary Table S3).

The mean maximum plasma nicotine concentration (C_max_) and area under the plasma nicotine concentration-time curve (AUC; the parameters AUC_0–90_, AUC_0–240_, and AUC_t_ were derived) values were highest for usual brand cigarettes and lower for each of the iSENZIA™ HHS flavor variants (Table 1). Statistical analyses showed that C_max_ and AUC_t_ values for iSENZIA™ HHS *Forest Berry* and *Summer Watermelon* variants were significantly lower than for subjects’ usual brand cigarettes but not significantly different from one another (Supplementary Table S4).

**TABLE 1:** Baseline-adjusted plasma nicotine pharmacokinetic parameters following controlled product use in the outcomes population of the clinical study.

	Variable	C_max_ (ng/mL)	AUC_0–90_ (ng*min/mL)	AUC_0–240_ (ng*min/mL)	AUC_t_ (ng*min/mL)	T_max_ (minutes)
Study product	n	25	25	25	25	25
iSENZIA™ HHS *Forest Berry*	Geometric mean	5.296	186.7	342.8	343.0	NC
	Geometric CV (%)	53.9	36.4	36.9	36.9	NC
	Mean	6.0	197.8	363.7	363.9	6.47
	SD	3.28	65.95	123.73	123.83	2.319
	Median	4.912	196.3	358.7	358.8	6.033
	Range	1.99, 16.7	107, 314	193, 561	193, 561	4.02, 13.75
iSENZIA™ HHS *Summer Watermelon*	Geometric mean	6.164	194.8	357.1	357.3	NC
	Geometric CV (%)	51.3	38.9	39.2	39.2	NC
	Mean	6.87	208.3	382.2	382.5	6.46
	SD	3.24	78.45	144.94	144.95	2.240
	Median	6.247	178.3	372.7	373.5	6.067
	Range	2.43, 14.8	97.9, 407	172, 736	172, 736	3.98, 15.32
Usual brand cigarettes	Geometric mean	16.44	620.6	1077	1078	NC
	Geometric CV (%)	51.9	35.9	35.5	35.5	NC
	Mean	18.5	658.7	1143.0	1144.0	7.1
	SD	10.05	242.87	427.68	427.87	1.66
	Median	16.48	628.6	1039	1042	6.350
	Range	5.38, 51.1	317, 1330	616, 2200	617, 2210	4.02, 10.25

Abbreviations: AUC_0–90_ and AUC_0–240_, area under the plasma nicotine concentration-time curve from zero to 90 min and 240 min minutes, respectively; AUC_t_, area under the plasma nicotine concentration-time curve from zero to the time of the last measurable non-zero concentration; C_max_, maximum plasma nicotine concentration; CV, coefficient of variation; HHS, heated herbal system; n, number of observations used in the analysis; NC, not calculated; SD, standard deviation; T_max_, time of the maximum plasma nicotine concentration.

Among the iSENZIA™ HHS flavor variants, the median time taken to reach the maximum plasma nicotine concentration (T_max_; Table 1) was highest for subjects’ usual brand cigarettes (6.350 min), and marginally lower for the iSENZIA™ HHS *Forest Berry* (6.033 min) and *Summer Watermelon* (6.067 min). Median T_max_ for subjects’ usual brand cigarettes was 6.250 min (Table 1). Statistical analyses of differences in T_max_ between the study products showed that these were not statistically significant for any of the comparisons (Supplementary Table S5).

#### Subjective effects

The urge to smoke was assessed prior to study product use and at various time points after product use using a single-item questionnaire. Subjects responded to the question “Right now, how much would you like to smoke a cigarette?” by providing responses on a 100 mm visual analog scale (VAS) ranging from “Not at all” to “A great deal.” Mean baseline VAS scores ranged from 90.0 to 92.9 across all products. Use of all study products elicited robust reductions in urge to smoke (Figure 4), which were greater for subjects’ usual brand cigarettes (Figure 4). The mean maximum change in urge to smoke (E_max_) was highest for subjects’ usual brand cigarettes (69.2 ± 28.38) and approximately 36%–38% lower for the iSENZIA™ HHS flavor variants (Table 2). E_max_ was not significantly different between the iSENZIA™ HHS flavor variants, but the differences between the iSENZIA™ HHS flavor variants and usual brand cigarettes were significant (Supplementary Table S6). The time of the maximum change in urge to smoke (TE_max_) was highest for iSENZIA™ HHS *Forest Berry*, intermediate for iSENZIA™ HHS *Summer Watermelon*, and lowest for usual brand cigarettes (Table 2). Mean AUEC_0–240_ (area under the effect-time curve from zero to 240 min) was highest for subjects’ usual brand cigarettes, and significantly lower for both iSENZIA™ HHS flavor variants (Table 2; Supplementary Table S6). However, no statistically significant differences in AUEC_0–240_ were seen between the individual iSENZIA™ variants.

**TABLE 2:** Summary of urge to smoke parameters following controlled product use in the outcome population of the clinical study.

Parameter	n	iSENZIA™ HHS *Forest Berry*	iSENZIA™ HHS *Summer Watermelon*	Usual brand cigarettes
		25	25	25
E_max_	Mean ± SD	43.0 ± 31.70	44.3 ± 28.87	69.2 ± 28.38
	Median	38.0	41.0	80.0
	95% CI	29.9, 56.0	32.4, 56.2	57.5, 80.9
TE_max_	Mean ± SD	21.6 ± 47.62	14.1 ± 17.52	11.1 ± 12.93
	Median	7.100	7.050	7.217
	95% CI	1.92, 41.24	6.83, 21.29	5.71, 16.39
AUEC_0–240_	Mean ± SD	3369 ± 3720.2	3228 ± 3892.8	6083 ± 6037.4
	Median	2444	2010	6579
	95% CI	1833, 4905	1622, 4835	3591, 8575

Abbreviations: AUEC_0–240_, area under the effect-time curve from zero to 240 min minutes; CI, confidential interval; E_max_, maximum value of the difference between pre- and post-use; HHS, heated herbal system; n, number of observations; SD, standard deviation; TE_max_, time of the maximum difference between pre- and post-use.

Product evaluation scale factor scores are presented in Table 3. On a scale of 1–7, with 1 being “not at all” and 7 being “extremely,” overall mean product evaluation scores for iSENZIA™ HHS *Forest Berry* and *Summer Watermelon* in the domain of satisfaction were 3.5 ± 1.61 and 3.7 ± 1.55, respectively, with a higher score of 5.8 ± 1.31 for subjects’ usual brand cigarettes. Similarly, in the domains of psychological reward, relief, ease of use, comfort using in public, and concerns about dependence, mean values were highest for subjects’ usual brand cigarettes and marginally lower, and comparable, for each of the iSENZIA™ HHS flavor variants (Table 3). For aversion, mean values were similar across all study products.

**TABLE 3:** Product evaluation scale factor scores by study product in the outcome population of the clinical study.

Subscale	n	iSENZIA™ HHS *Forest Berry*	iSENZIA™ HHS *Summer Watermelon*	Usual brand cigarettes
		25	25	25
Satisfaction	Mean ± SD	3.5 ± 1.61	3.7 ± 1.55	5.8 ± 1.31
	Median	3.3	3.5	6.0
	95% CI	2.80, 4.13	3.02, 4.30	5.21, 6.29
Psychological reward	Mean ± SD	2.9 ± 1.37	3.4 ± 1.30	5.2 ± 1.19
	Median	2.80	3.20	5.00
	95% CI	2.38, 3.51	2.85, 3.92	4.68, 5.67
Aversion	Mean ± SD	1.3 ± 0.41	1.3 ± 0.70	1.4 ± 0.78
	Median	1.00	1.00	1.30
	95% CI	1.17, 1.50	1.04, 1.62	1.12, 1.76
Relief	Mean ± SD	3.1 ± 1.43	3.3 ± 1.42	5.4 ± 1.23
	Median	2.80	3.00	5.60
	95% CI	2.48, 3.66	2.72, 3.89	4.86, 5.88
Was it easy to use?	Mean ± SD	5.6 ± 1.58	6.0 ± 1.12	6.4 ± 1.04
	Median	6.0	6.0	7.0
	95% CI	4.9, 6.2	5.5, 6.5	5.9, 6.8
Comfortable using the product in public?	Mean ± SD	5.3 ± 1.34	5.4 ± 1.58	6.2 ± 1.27
	Median	5.0	6.0	7.0
	95% CI	4.7, 5.8	4.7, 6.1	5.7, 6.8
Concerned you would become dependent?	Mean ± SD	2.4 ± 1.61	2.2 ± 1.59	3.6 ± 2.12
	Median	2.0	1.0	4.0
	95% CI	1.7, 3.1	1.6, 2.9	2.7, 4.5

Subscale scores in the domains of satisfaction (items 1, 2, 3, and 12), psychological reward (items 4, 5, 6, 7, and 8), aversion (items 9, 10, 16, and 18), and relief (items 11, 13, 14, 15, and reversed for 20) were generated from the individual items as described previously (Hatsukami et al., 2013). Abbreviations: CI, confidence intervals; HHS, heated herbal system; n, number of observations; SD, standard deviation.

Regarding intent to use the product again, 52% and 44% of subjects expressed positive likelihood (assessed as a rating on the VAS between the midpoint and “Extremely”) of buying and using the iSENZIA™ HHS *Forest Berry* and *Summer Watermelon*, respectively, compared with 76% of subjects who expressed positive likelihood of using their usual brand cigarettes again (Table 4). Overall, the mean raw VAS scores for intent to use the product again were 43.4 and 44.4 (0 = definitely would not and 100 = definitely would) for iSENZIA™ HHS *Forest Berry* and *Summer Watermelon*, respectively, with a higher mean score of 71.2 for subjects’ usual brand cigarettes.

**TABLE 4:** Intent to use iSENZIA^™^ heated herbal systems (HHS) compared to usual brand cigarettes in the outcomes population of the clinical study.

Bipolar score category	Sex	iSENZIA™ HHS Forest Berry	iSENZIA™ HHS *Summer Watermelon*	Usual brand cigarettes
		25	25	25
−50 to <0	Male	8 (32%)	8 (32%)	3 (12%)
	Female	4 (16%)	6 (24%)	3 (12%)
	Overall	12 (48%)	14 (56%)	6 (24%)
0	Male	0 (0%)	0 (0%)	0 (0%)
	Female	0 (0%)	0 (0%)	0 (0%)
	Overall	0 (0%)	0 (0%)	0 (0%)
>0 to 50	Male	7 (28%)	7 (28%)	12 (48%)
	Female	6 (24%)	4 (16%)	7 (28%)
	Overall	13 (52%)	11 (44%)	19 (76%)

Intent to use was measured using a 100-point visual analog scale (VAS), where 0 indicated “definitely would not use again” and 100 indicated “definitely would use again.” A positive likelihood of use was defined as a score above the midpoint (>50). The table includes the frequency of intent to use VAS, bipolar scores stratified by sex and product, and the percentage of subjects expressing intent to use. Abbreviations: HHS, heated herbal system.

#### Safety

There were no serious adverse events (SAEs) reported in this study, and no subjects discontinued due to adverse events (AEs). Overall, AEs were infrequently reported in this study, with AEs reported by five (20%) and three (12%) subjects associated with the use of iSENZIA™ HHS *Forest Berry* and *Summer Watermelon*, respectively. Mild dizziness was the most frequently reported AE, with eight (32%) subjects reporting dizziness events, including five (20%) subjects following use of their usual brand cigarettes, three (12%) subjects following iSENZIA™ HHS *Summer Watermelon* use, and two (8%) subjects following iSENZIA™ HHS *Forest Berry* use. Mild nausea was reported by two (8%) subjects following iSENZIA™ HHS *Summer Watermelon* use and one (4%) subject following either iSENZIA™ HHS *Forest Berry* or usual brand cigarette use.

Catheter site pain was reported three times by three (13%) subjects, and the remaining AEs (constipation, dizziness, and neck pain) were reported by one (4%) subject each. The constipation and dizziness events were moderate in severity, and the catheter site pain and neck pain events were mild. The investigator considered all AEs to be unlikely related or unrelated to the study product.

Mean vital signs (heart rate and blood pressure) remained within normal limits at all study time points, with minimal change from baseline. There were no individual clinically significant vital sign findings in this study.

## Discussion

In this article, we describe the findings from a suite of pre-clinical and clinical studies assessing the iSENZIA™ HHS with two flavor variants. The HHS heats a non-tobacco, nicotine-containing tea-based substrate to produce an inhalable aerosol. Our main pre-clinical findings were that the iSENZIA™ HHS aerosols did not contain six of the WHO TobReg 9 analytes at levels above their respective limits of quantification. Those that were detectable were found at levels substantially lower than the levels found in 1R6F reference cigarette smoke, by up to approximately 99.8%. These aerosol toxicant reductions translated into significant reductions in biological activity in three toxicological assays assessing cytotoxicity, mutagenicity, and genotoxicity compared to 1R6F cigarette smoke. In a clinical study among adult cigarette smokers, both flavor variants of the iSENZIA™ HHS delivered nicotine and elicited subjective effects such as satisfaction, psychological reward, and relief, but to a lesser degree than subjects’ usual brand cigarettes. This is overall suggestive that the iSENZIA™ HHS may offer a harm-reduced, acceptable, and satisfying alternative to cigarettes among smokers with a lower abuse liability.

Our studies are the first to report aerosol chemistry and *in vitro* toxicology of an HHP, and as such, we cannot corroborate our findings with those of other studies in the literature. However, the pre-clinical study findings are consistent with those in the literature for HTPs, which similarly use electrical heating of consumable sticks but with a tobacco-based substrate. Similar to the iSENZIA™ HHS, the aerosol generated from HTPs contains either lower levels of, or an absence of, HPHCs and other toxicants when compared to cigarette smoke (Chapman et al., 2023a; Forster et al., 2018; Jaccard et al., 2018; Mallock et al., 2018; Farsalinos et al., 2018; Schaller J.-P. et al., 2016; Hashizume et al., 2023). For those toxicants present in HTP aerosols, the percentage reductions compared with reference cigarette smoke are comparable to those reported here for the iSENZIA™ HHS (Chapman et al., 2023a; Schaller J.-P. et al., 2016; Hashizume et al., 2023). Of particular importance, the very low level of CO in both the iSENZIA™ HHS and HTP aerosols is indicative of a lack of combustion during heating of both the tea- and the tobacco-based consumable sticks (Chapman et al., 2023a; Schaller J.-P. et al., 2016; Hashizume et al., 2023). More generally, the toxicant reductions common to both product types are indicative of the potential for reduced exposure among smokers switching to exclusive use of either product type, which has previously been demonstrated for HTPs in clinical biomarker studies (Gale et al., 2021a; Gale et al., 2021b; Gale et al., 2019; Gale et al., 2022; Haziza et al., 2020a; Haziza et al., 2016; Haziza et al., 2017; Lüdicke et al., 2018a). Perhaps of note regarding differences in toxicant levels between the iSENZIA™ HHS and HTP aerosols is the absence of tobacco-specific nitrosamines (TSNAs: NNN and NNK) in the iSENZIA™ HHS aerosol. TSNAs are present at low levels in aerosols from some HTPs (Jaccard et al., 2017; Chapman et al., 2023a; Forster et al., 2018; Schaller J.-P. et al., 2016; Hashizume et al., 2023), although these levels are much lower than those in cigarette smoke. Similarly, B[a]P, formaldehyde, 1,3-butadiene, and benzene were below their respective limits of quantification in iSENZIA™ HHS aerosols, but some or all of these toxicants have been reported at low levels in some HTP aerosols (Chapman et al., 2023a; Forster et al., 2018; Mallock et al., 2018; Farsalinos et al., 2018; Schaller J.-P. et al., 2016; Hashizume et al., 2023). The percentage reductions in acrolein and acetaldehyde in the iSENZIA™ HHS aerosols compared to reference cigarette smoke appear greater than the percentage reductions reported for both IQOS and a prototype HTP (Farsalinos et al., 2018; Schaller J.-P. et al., 2016; Hashizume et al., 2023). To summarize for iSENZIA™ HHS aerosol: Both HHS products deliver non-quantifiable amounts of six of nine priority compounds (NNN, NNK, BaP, 1,3-butadiene, benzene, and formaldehyde) in aerosol under the stated puffing conditions conditions. The remaining three substances of the TobReg 9 analytes (CO, acetaldehyde, and acrolein) could be quantified but are still reduced by >96% on a per-puff basis compared to the reference cigarette 1R6F. We acknowledge that only a limited number of analytes were presented here. A broader range of analytes, combined with non-targeted analysis of the aerosols, is required to fully characterize the chemistry of HHP aerosols and will form the basis of future publications.

The iSENZIA™ HHS aerosol toxicant reductions relative to 1R6F cigarette smoke resulted in reduced biological activity of the iSENZIA™ HHS whole aerosol when assessed using assays to detect potential cytotoxic, mutagenic, and genotoxic effects, which concurs with findings from *in vitro* toxicological studies assessing HTP aerosols (Chapman et al., 2023a; Schaller J.-P. et al., 2016; Hashizume et al., 2023; Keyser et al., 2024; Chapman et al., 2023b; Wang et al., 2021; Thorne et al., 2020; Thorne et al., 2018). This suggests that iSENZIA™ HHS use is potentially less harmful to users than cigarette smoking and provides initial evidence of its THR potential. While additional studies are required to confirm the risk reduction potential, it is notable that the reductions in aerosol toxicants and *in vitro* biological activity, described above for HTPs, which were either similar or lesser in magnitude than our findings for the iSENZIA™ HHS, led to reduced *in vivo* toxicological responses (Titz et al., 2020; Phillips et al., 2018; Titz et al., 2018; Kogel et al., 2016; Wong et al., 2016). As well as the reductions in biomarkers of exposure described above, clinical evidence from studies assessing the impact of exclusive switching to HTP use is also associated with favorable changes in a number of biomarkers of potential harm (Gale et al., 2022; Haziza et al., 2020b; Lüdicke et al., 2019; Lüdicke et al., 2018b). Overall, while further studies are required, our findings support the idea that, at the level of the individual smoker, the iSENZIA™ HHS has THR potential.

THR is based on the principle that the individual- and population-level harms associated with cigarette smoking can be reduced by providing adult smokers with smoking alternatives that deliver nicotine but with levels of HPHCs and other toxicants that are either reduced or absent (Institute of Medicine, 2001; Stratton et al., 2001). The pre-clinical study findings described above provide evidence of the potential for exposure reductions among smokers who fully switch to using the iSENZIA™ HHS, and therefore the potential for reduced human health impact compared to cigarette smoking. Our clinical study findings additionally suggest that adult smokers are likely to find the iSENZIA™ HHS to be an acceptable and satisfying alternative to cigarettes, which is an important determinant of whether a given product will support smokers, who would otherwise continue to smoke, to transition away from cigarette smoking. Two major components thought to contribute to acceptability are nicotine delivery and the elicitation of positive subjective effects such as satisfaction, reward, and relief from smoking urges and withdrawal symptoms; both factors were demonstrated in the clinical study. Overall, as suggested for other smoking alternatives such as HTPs and e-cigarettes, these attributes can contribute to the uptake of alternatives to cigarette smoking, such as the iSENZIA™ HHS, among adult smokers, its continued use, and the prevention of relapse back to cigarette smoking (Fearon, 2022; Goldenson et al., 2021; Hajek et al., 2020; Phillips-Waller et al., 2021a; Phillips-Waller et al., 2021b; Hughes, 2007; Lechner et al., 2018; Shiffman and Kirchner, 2009; West, 2017).

It is important to consider other factors that may determine the overall THR potential of smoking alternatives. Abuse liability, also termed dependence potential, is a multifactorial measure that provides an estimation of the likelihood that use of a tobacco product will give rise to dependence on its use. Abuse liability can be determined by considering findings from nicotine pharmacokinetic and subjective effect assessments, which together constitute an assessment of abuse liability (Fearon, 2022; Vansickel et al., 2022). The THR potential of novel nicotine and tobacco products is proposed to be maximized when appeal and abuse liability are high and when toxicity/harmfulness are low (Abrams et al., 2018). Novel products that possess at least some degree of abuse liability may be beneficial because this allows the novel product to compete with cigarettes and support transitioning away from smoking (Abrams et al., 2018; Fearon, 2022; Ashley et al., 2020; Cahn et al., 2021). However, high abuse liability may lead to unintended consequences because the novel product may pose an initiation or addiction risk among non-users of nicotine products, particularly among susceptible populations such as youth and young adults (Ashley et al., 2020; Cahn et al., 2021). In this regard, optimizing abuse liability may be regarded as a balancing act between maximizing uptake and exclusive use among the intended audience, that is, adult cigarette smokers, while minimizing use among those for whom use is not intended (never smokers, ex-smokers, and youth). Cigarettes have the highest abuse liability of any tobacco products due to their ability to rapidly and efficiently deliver nicotine into the blood of smokers and their elicitation of strong subjective effects, while forms of nicotine delivery systems that deliver nicotine more slowly and less substantially have a lower abuse liability (Abrams et al., 2018; Gottlieb and Zeller, 2017; Carter et al., 2009). We concluded from our clinical study findings that the iSENZIA™ HHS has a lower abuse liability than cigarettes because, although both flavor variants of the iSENZIA™ HHS robustly delivered nicotine and elicited positive subjective effects among users, the magnitudes of these effects were lower than those observed when subjects smoked their usual brand cigarettes. Whilst this should not be interpreted as suggesting that iSENZIA™ HHS use is not addictive, it is suggestive that its use will likely have lower positive reinforcing effects. In the context of population-level THR, this finding supports that while the iSENZIA™ HHS may be an acceptable alternative to cigarette smoking that is satisfying to adult smokers and therefore may support transitioning away from smoking, the lower abuse liability relative to cigarettes suggests that iSENZIA™ likely presents less of an initiation/addiction risk among non-users of nicotine. Overall, this potential generation of an “off-ramp” away from cigarette smoking, while minimizing the “on-ramp” to initiating tobacco/nicotine use, may enhance the contribution the iSENZIA™ HHS can make to THR strategies.

The results presented in this article should be considered in the context of several limitations. First, our studies only assessed a single type of HHP, and our data may not be extrapolated to other HHPs that may either use different types and compositions of non-tobacco substrates or heat via different means and temperatures. We also only assessed two of the available iSENZIA™ HHS flavor variants. Second, our pre-clinical studies generated aerosol from the iSENZIA™ HHS using a single puffing regimen, and this may not be indicative of puffing patterns of users in the real world. However, to date, no data are available concerning puffing topography among users of HHPs. Furthermore, our pre-clinical analytical chemistry and toxicology studies were limited to a small number of endpoints and further chemical and biological characterization of the iSENZIA™ HHS aerosol is necessary in order to fully understand the potential human health impact of iSENZIA™ HHS use, and human exposure and/or biological effects studies would also be informative in this regard. Third, our clinical study has a number of limitations, including that we only assessed nicotine pharmacokinetics and subjective effects profiles. While the findings from these studies support the conclusion that iSENZIA™ HHS use may provide an acceptable alternative to smoking cigarettes, no data were presented concerning its actual ability to help smokers transition away from cigarette smoking. Additionally, and similar to the pre-clinical limitation described earlier, the clinical study assessed the use of the iSENZIA™ HHS in a clinical laboratory environment using a controlled puffing regimen, and also at a single point in time with only a very brief familiarization period. While this experimental approach allows us to estimate the relative abuse liability of the iSENZIA™ HHS compared to cigarettes, absolute abuse liability in real-world settings may differ from that reported in the clinical study.

In conclusion, our pre-clinical studies provide evidence that the aerosol from the iSENZIA™ HHS is fundamentally different from cigarette smoke and contains significantly lower levels of HPHCs and NNN, all of which were mandated for lowering in cigarette smoke in the WHO TobReg proposal. These lower levels of toxicants give rise to a significantly reduced biological impact in a number of *in vitro* toxicological assays. Because the iSENZIA™ HHS delivered satisfactory levels of nicotine to users and generated positive subjective effects and reduced urges to smoke in a clinical study, it may provide a suitable alternative to smoking for adults who would otherwise continue to smoke. The abuse liability of the iSENZIA™ HHS is likely to be lower than that of cigarettes, which may help to limit its ability to facilitate initiation among non-users of tobacco and nicotine products. Overall, considering the totality of data presented, the iSENZIA™ HHS is a potentially reduced exposure and risk alternative to cigarettes that may play a meaningful role in THR by generating an “off-ramp” away from cigarette smoking for adult smokers while minimizing the “on-ramp” to initiation of tobacco and/or nicotine use. Further studies, however, must be conducted to fully understand the potential human health impact of iSENZIA™ HHS use and its contribution to THR. These studies should assess mechanistic toxicological impacts, broader clinical assessments reflecting real-world usage, and long-term behavioral outcomes.

## Methods

### Products assessed in pre-clinical and clinical studies

#### iSENZIA™ heated herbal system

The iSENZIA™ HHS, which comprises the PULZE™ 2.0 heating device and iSENZIA™ sticks (see Supplementary Figure S1), generates an inhalable aerosol by heating the sticks using an electrically powered heating device. Once switched on, the PULZE™ 2.0 heating device is operational for 5 min, which allows users to take approximately 10 puffs from a single iSENZIA™ stick. The device can be operated in two different user-selected modes (Intense and Mild modes) in which the device heats to temperatures below combustion. In the studies described in this article, the iSENZIA™ HHS was used in the Intense mode.

The iSENZIA™ sticks contain a portion of substrate that is tea-based and contains other components, including cellulose, nicotine, a binder, and a variety of flavor ingredients. Two flavored iSENZIA™ consumables (used with the PULZE™ 2.0 heating device) were assessed in the pre-clinical and clinical studies described: iSENZIA™ *Forest Berry* and iSENZIA™ *Summer Watermelon*. These flavors are released into the tea-based substrate prior to heating by squeezing a capsule (“crushball;” see Supplementary Figure S1) that contains the flavoring, as per the standard procedure and according to the study protocol. The tea-based substrate in each of the iSENZIA™ sticks had a target specification of 2.7 mg of nicotine per stick.

#### Cigarettes

The 1R6F reference cigarette was used as a comparator product in the pre-clinical analytical chemistry and *in vitro* toxicology studies (CTRP, 2023). These cigarettes were stored frozen and unopened in their original packaging until conditioned. In the clinical study, all subjects provided their usual brand of cigarette for use as a comparator product.

### Pre-clinical studies

#### Emissions chemistry

##### Cigarette smoke and iSENZIA™ HHS aerosol generation

The 1R6F reference cigarettes were smoked on a rotary smoking machines (RM20D, RM 400A2, and RM 20H2, Borgwaldt (Körber), Hamburg, Germany) according to the ISO 20778:2018 smoking regimen (55 mL puff volume, 2-s puff duration, 30-s puff interval, bell-shaped puff profile, filter vents blocked). The iSENZIA™ HHS was used on a linear smoking machine (LM4C Borgwaldt (Körber), Hamburg, Germany) according to a modified version of ISO 20778:2018 (55 mL puff volume, 2-s puff duration, 30-s puff interval, bell-shaped puff profile, with no filter vent blocking). The filter vent must be blocked if there is a possibility that the vent could be covered while smoking. As iSENZIA™ HHS does not have ventilated filters, filter blocking was not required during testing. The aerosol was trapped on either a Cambridge filter pad in a gas collection bag (e.g., Tedlar bag) or an impinger using 2,4-Dinitrophenylhydrazine (DNPH) solution, depending on the targeted analytes.

Individual analytes assessed were those nine listed in the WHO TobReg proposal (Burns et al., 2008), plus aerosol-collected mass (ACM)/total particulate matter (TPM) and nicotine. To determine ACM and TPM, iSENZIA™ HHS whole aerosol or 1R6F reference cigarette whole smoke was trapped on a Cambridge filter pad. The mass of the filter pad, including the holder, was determined before and after use, and the mass of the collected matter per iSENZIA™ HHS stick and per 1R6F reference cigarette was deemed to be the ACM and TPM, respectively.

For the extraction and measurement of TSNAs, Cambridge filter pads (92 mm diameter) following machine smoking of 1R6F reference cigarettes were transferred to a 100-mL Erlenmeyer flask containing 40 mL of extraction solution (1:1 methanol: water, containing deuterated internal standard compounds). Cambridge filter pads (44 mm diameter) following machine use of the iSENZIA™ HHS were transferred to a 50-mL Erlenmeyer flask containing 20 mL of extraction solution. The flasks were shaken using an automated shaker for 60 min at ∼180 rpm. Following this extraction period, aliquots of the extraction solutions were membrane filtered into vials for liquid chromatography-tandem mass spectrometry (LC-MS/MS) analysis to determine levels of NNN and NNK. For the high-performance liquid chromatography (HPLC), an Agilent 1290 system was used with a Gemini 3 µm C18 column (150 mm × 4.6 mm, 110 Å) (55°C), injection volume: 5 μL, flow rate: 800 µL/min. The eluent gradient was applied as follows: 0 min, 40% A (0.05% acetic acid in water):60% B (0.05% acetic acid in methanol); 4 min, 40% A:60% B; 5 min, 90% A:10% B; 6 min, 90% A:10% B. An API 6500 QTRAP (SCIEX, Framingham, MA, United States) was used for the MS/MS step: Turbo Spray ion source, ESI positive ionization mode; 4,500 V; 500°C; nebulizing gas pressure, 70 psi; turbo heater gas pressure, 70 psi; dwell time 60 ms. Multiple reaction monitoring (MRM) quantification was performed using NNN-d4 and NNK-d4 (purity >98%; TRC, ON, Canada).

For measurement of B[a]P, the Cambridge filter pads were transferred to 100 mL Erlenmeyer flasks and extracted for 20 min with 30 mL cyclohexane containing the internal standard, B[a]P-d12 (Dr. Ehrenstorfer, LGC). Aliquots of 15 mL were then reduced to a volume of 3 mL under reduced pressure using a Turbovap apparatus. The samples were cleaned using an NH2-Phase solid phase extraction (SPE) column with 500 mg of sorbent (Phenomenex, CA, United States), reduced to a final volume of 0.5 mL, and then analyzed using gas chromatography-mass spectroscopy (GC-MS). For the GC-MS, a Thermo Trace 1310 GC system with a Thermo TSQ 8000 Evo MS detector was used along with a J&W DB-17 ms GC-capillary (30 m, 0.25 mm, 0.25 µm). The following GC temperature program was applied: initial temperature: 120°C, hold for 1 min, ramp 30°C/min to 310°C, hold for 20 min. A flow rate of 1 mL/min (helium) was set; injector temperature: 300°C; injection volume: 1 µL splitless; MS source temperature: 300°C; transfer line temperature: 290°C. Detection mode: selected ion monitoring, B[a]P: 252.1, 250.1 and B[a]P-d12: 264.1, 260.1.

For carbonyl quantification, the unfiltered mainstream smoke/aerosol was guided through two impingers containing 35 mL 2,4-dinitrophenylhydrazine (DNPH) solution (>99.9% purity, Merck). Following smoking, the contents of the two impingers were combined and transferred to Erlenmeyer flasks. Here, the carbonyl derivatization reaction was carried out for 30 min, following which time aliquots were taken and stabilized using Trizma base solution (Sigma Aldrich, Germany) and membrane filtered prior to analysis via high-performance liquid chromatography with diode-array detection (HPLC-DAD). For this, an Agilent 1100 Series HPLC-DAD was used with an RP C18 column (125 Å, 150 mm × 4.6 mm, 3 µm) (Phenomenex, CA, United States); sample injection volume, 20 μL; column oven temperature, 40°C; flowrate, 1.2 mL/min; 365 nm detector. The eluent gradient was applied as follows: 0 min, 60% A (acetonitrile:tetrahydrofuran:isopropanol:H_2_O 30:10:1:59): 40% B (acetonitrile:H_2_O 65:35): 0% C (acetonitrile); 12 min, 60% A: 40% B: 0% C; 15 min, 60% A: 40% B: 0% C; 21 min, 0% A: 100% B: 0%C; 22.2 min, 0% A: 0% B: 100% C; 25.2 min, 0% A: 0% B: 100% C; 28.2 min, 100% A: 0% B: 0% C; 34.2 min, 100% A: 0% B: 0% C. For nicotine, the particulate phase of the HHS aerosol was trapped on a Cambridge filter pad, and the filter pad was extracted with propan-2-ol. An aliquot was analyzed using a GC-flame ionization detector (FID).

For the gas phase analytes, the vapor phase of the HHS aerosol was collected in a Tedlar bag located after the Cambridge filter pad. Vapor phases containing the volatiles selected for analysis were injected directly into a GC 7890 A (Agilent Technologies) with a DB 624 UI (60 m, 0.25 mm, 1.4 µm) column and an MS 5975 C detector (Agilent Technologies) with a Loop Filling Manager 205 (Teutner Analysentechnik GmbH, Germany). GC oven temperature: 40°C for 6 min, heating rate 20°C/minute, 230°C for 5 min; run time, 20.5 min. The injector temperature was 180°C; split, 1:20; constant flow rate, 1.5 mL/min, and the valve box temperature was 180°C. The MS source temperature was 230°C; MS Quad, 150°C; transfer line, 230°C; detection mode: selected ion monitoring. The sample (vapor phase) was separated by gas chromatography (GC), and selected gas phase compounds were quantified by mass spectrometry (MS). Identification of the single components was carried out using the retention time and their specific masses. For CO, the vapor phase of the aerosol was also collected after passing through a Cambridge filter pad. The CO content was determined using a calibrated non-dispersive infrared analyzer (NDIR).

Where appropriate, limits of quantification for the analytes were as follows: NNN, 5 ng; NNK, 5 ng; B[a]P, 2.5 ng; formaldehyde, 1.58 µg; 1,3-butadiene, 0.6 µg; acetaldehyde, 10.5 µg; acrolein, 2.63 µg; benzene, 0.27 µg. All values are per stick or per cigarette.

### *In Vitro* toxicology

#### Cigarette smoke and iSENZIA™ HHS aerosol generation

Prior to the generation of cigarette smoke, the 1R6F reference cigarettes were conditioned according to ISO 3402:1999 (ISO 3402:1999, 1999), while the iSENZIA™ HHS sticks were not conditioned. For the NRU and IVM assays, the fresh smoke/aerosol was generated using the Smoke Aerosol Exposure *In Vitro* System (SAEIVS; Burghart Tabaktechnik, Wedel, Germany) (Wieczorek et al., 2023). Generation of both cigarette smoke and aerosol was performed in accordance with the ISO 20778:2018 smoking regimen (55 mL puff volume, 2-s puff duration, 30-s puff interval, bell-shaped puff profile) (ISO 20778:2018, 2018). For the Ames assay, bacterial cultures were exposed to the whole smoke/aerosol using a Smoking Robot VC 10-S (Vitrocell Systems GmbH, Waldkirch, Germany). Generation of fresh smoke from 1R6F reference cigarettes (CTRP, 2023) was performed according to the ISO3308:2012 smoking regimen (35 mL puff volume, 2 s puff duration, 60 s puff interval, bell-shaped puff profile) (International Organization for Standardization, 2012) while for the iSENZIA™ HHS, ISO 20778:2018 (ISO 20778:2018, 2018) without vent blocking was used to generate the aerosol.

#### Neutral red uptake cytotoxicity assay

Cytotoxicity of the whole smoke/aerosols was measured using the NRU assay with Beas-2B human bronchial epithelial cells (ECACC Catalogue No. 95102433). Cell stocks were stored in liquid nitrogen until use, and cultures were checked for the absence of *mycoplasma*. The Beas-2B cells were cultured in Epithelial Cell Growth Medium (Promocell #C-21060) with Supplement Mix (Promocell #C-39165) added. Only cells between 3 and 20 passages after thawing were used for the experiments. Prior to whole smoke/aerosol exposure, 100 μL of cell suspension was seeded at a cell density of 0.5 × 10^4^ cells/mL into the inner 60 wells of 96-well round-bottomed collagen I-coated plates and incubated at 37°C and 5% CO_2_ for 20 ± 3 h. The collagen I-coated plates were prepared by adding 25 μL of collagen I solution (20% PureCol^®^ EZ Gel, 2% 1 M HEPES buffer, and 78% cell culture medium; final collagen I concentration, 0.1%) to each well of the 96-well plate. Directly prior to exposure, medium was removed from the cells by suction and reverse plate centrifugation for 10 s at 70 × *g* to guarantee complete and homogenous removal of medium from the wells for the ALI exposures. Cells were then exposed at the ALI to increasing puff numbers of fresh whole smoke/aerosol using the SAEIVS according to the puffing regimens described above. The final exposure range for the positive control of 1R6F smoke (diluted either 1:15 or 1:20 with air) was 0–0.8 puffs (corrected for dilutions) and 0–20 puffs for the test item HHP aerosols (undiluted). Cells were exposed for not longer than 15 min, and following exposure, 200 μL of fresh medium was added to each well and cells were incubated for 65 ± 2 h. Following this incubation period, the incubation medium was replaced by 200 μL fresh medium containing neutral red dye for 3 h, during which time the dye was taken up by viable cells. After washing and lysing of the cells, absorbance (540 nm) in the wells was then measured using a Tecan Sunrise plate reader, with absorbance directly proportional to the number of live cells present. From the absorbance values, the mean relative cell viability compared to the values measured for control wells (0 puffs of smoke/aerosol) was calculated for each test concentration. Three replicates were performed on different days. Each day, two 96-well multiwell plates were exposed, each with six wells exposed to the same number of pulls.

#### Ames bacterial reverse mutation genotoxicity assay

Mutagenic potential was determined using the *in vitro* bacterial reverse mutation test (Ames assay) with *Salmonella Typhimurium* strains TA98 and TA100 (+/−S9; Trinova Biochem GmbH, Giessen, Germany) in compliance with the Organisation for Economic Co-operation and Development (OECD) Test Guideline 471 (Organisation for Economic Co-operation and Development, 2020). Nutrient broth No. 2 (OXOID) cultures were prepared by inoculating 40 mL of medium with 0.7 mL of 6 h pre-culture in a 100-mL Erlenmeyer flask. These were incubated overnight at 37°C and shaken at 120 rpm. Following this, the respective bacterial suspensions were pooled into 120 mL suspensions (3 × 40 mL) and centrifuged at 1800 × *g* for 10 min. The supernatant medium was removed, and cells were resuspended in 12 mL of Dulbecco’s Ca^2+^- and Mg^2+^-free PBS (DPBS). A 10-mL sample of this suspension was added to a glass tube, which was inserted into an impinger connected to the Vitrocell VC 10-S Smoking Robot. Exposure to the test article smoke/aerosols was carried out at room temperature and protected from direct light.

The bacterial suspensions were exposed to increasing puff numbers of undiluted fresh whole smoke/aerosol. The final exposure range for the 1R6F smoke was 70 puffs, and it was 120–200 puffs for the HHS aerosols. During exposure, 350 μL of bacterial suspension was taken at regular intervals. For each repeat (Petri dish), 50 μL of the suspension was added to sterile 15 mL tubes, followed by 0.5 mL of 5% S9 mix or 0.5 mL 0.2 M phosphate buffer, followed by 2 mL of top agar (45°C). This mixture was then poured onto Vogel–Bonner agar plates (3 plates +S9, three plates −S9 per biological replicate), and the top agar was distributed by tilting/rotating. When the top agar solidified, plates were inverted and incubated at 37°C for 48 h. Following this, the total number of revertant colonies per plate was counted automatically using the Synbiosis ProtoCOL SR Automatic Colony Counter (Meintrup-DWS). Validity of the results was checked against the following criteria: mean negative control counts fell within the historical range, positive controls (the smoke of 1R6F research cigarette) induced clear increases in revertant colonies (+/−S9), no more than 5% of plates were lost due to contamination/other unforeseen circumstances. Any observed toxic effects (pinpoint colonies or reduction of revertant number) were excluded from the analysis. For each test item, two biological replicates (independent smoking sessions) were set up, each with three technical replicates per defined number of puffs.

### *In Vitro* micronucleus genotoxicity assay

These assays were carried out in accordance with OECD Test Guideline 487 (Organisation for Economic Co-operation and Development, 2023) with the exception that only long-term experiments in the absence of S9 and short-term experiments in the presence of S9 as the most relevant were conducted. Due to technical limitations, a short-term treatment in the absence of S9 was not applicable in parallel. V79 Chinese hamster lung fibroblast cell (ECACC 86041102) stocks were stored in liquid nitrogen until use, and cultures were checked to confirm the absence of *mycoplasma*. Only cells between 3 and 20 passages after thawing were used for the experiments. Cells were cultured in Dulbecco’s modified Eagle’s medium supplemented with 10% fetal bovine serum. Prior to exposure, 400 μL of the medium was added to each well of a 24-well plate, and polycarbonate Transwell inserts (0.4-μm pore membrane; 140620, Nunc) were added to these. Aliquots (250 μL) of V79 cell suspension were seeded at a density of 1 × 10^5^ cells/mL into each insert, and then the plates were incubated at 37°C and 5% CO_2_ for 20 ± 2 h. Directly prior to whole smoke/aerosol exposure, the inserts were transferred to fresh plates containing 250 μL of medium per well supplemented with HEPES buffer (final concentration, 20 mM), and the apical medium was removed. Whole smoke/aerosol exposures were subsequently carried out at the ALI, and cells were exposed to increasing puff numbers of whole smoke (diluted) or aerosol (undiluted), achieved using the sliding plate cover within the SAEIVS exposure chamber. The exposure range for the (Whole iSENZIA™ HHS aerosol was 0–30 puffs (+/-S9) (iSENZIA™ *Forest Berry*) and 0–40 puffs (iSENZIA™ *Summer Watermelon*) without dilution. For the 1R6F smoke, 0 to 1.11 puffs (−/+S9) (corrected for dilution of 1:9 with air) were applied.

Exposures were no longer than 20 min. Based on toxicity values, an adaptation of puff numbers from 20 to 40 puffs was applied in the replicate test for iSENZIA™ *Summer Watermelon*. For iSENZIA™ *Forest Berry*, the puff number of 30 showed good toxicity results in both replicate tests. Following exposure, inserts were transferred to plates containing 400 μL of fresh basal medium. For metabolic activation of whole smoke/aerosol components, S9 mix containing an S9 fraction derived from Aroclor 1254-treated male Sprague–Dawley rats (10% *v*/v S9 fraction, 90% v/v REGENSYS A, 1.3 mM NADP; Trinova Biochem GmbH) was added to the medium (final concentration, 1% S9), and 175 μL of this added to each insert immediately following exposure. Following a 3-h incubation, the S9 mix was removed from the cells, and 175 μL fresh medium was added. Cells were then incubated for a 20 ± 2 h recovery period. For exposed cultures without metabolic activation, 175 μL of fresh medium was added into the inserts immediately following exposure, and cells were incubated for 20 ± 2 h.

Following this incubation period, cells were detached from the inserts using Accutase^®^ and counted using the Scepter^™^ Cell Counter (Millipore) to determine cell density for microscope slide preparation and cell counts for relative cell count (RCC), relative population doubling (RPD, used as measure for the assessment), and relative increase in cell count (RICC) calculation to assess treatment-induced toxicity. Cell suspensions were fixed to slides by spinning at 380 × g for 5 min using a cytospin and applying further spin cycles for drying. Fixative solution was then applied to the slides (methanol:glacial acetic acid:37% formaldehyde:water 150:18.5:1:30.5), followed by one rinse in methanol, and slides were allowed to air dry. Prior to slide analysis, cells were stained with 1 μg/mL DAPI in mounting medium (VECTASHIELD, H-1000). Slides were analyzed using the automated Metafer system with a Z2 microscope (Zeiss). In each of the two tests performed per test condition, two replicate preparations per dose level with at least 1,000 cells each were evaluated for the occurrence of micronuclei (MN). That is, in sum more than 2,000 cells per dose level were evaluated using pre-programmed Metafer parameters (Metafer version 3.14.2), based on the criteria described previously (Chapman et al., 2023a; Fenech, 1993). For each test article, two biological replicates, each with two technical replicates were carried out for each of the +/−S9 treatments.

#### Statistical analyses

Typical dose–response curves for the neutral red uptake cytotoxicity assay follow a symmetrical sigmoidal shape and the effective concentration IC_50_ is defined as the concentration that causes a response halfway between minimum and maximum response. Thus, the IC_50_ describes the test substance concentration needed to achieve a 50% growth inhibition. The statistical evaluation and determination of the IC50 were carried out with the software GraphPad Prism 8.4.3. Based on the basal and maximum response, as well as the Hill slope, a nonlinear regression model calculates the IC50 value with the associated confidence intervals. The Hill slope describes the steepness of the curve and is calculated individually for each data set.

The Ames assay was considered valid if the mean negative control colony count fell within the normal/historical range and the positive control induced a clear increase in revertant numbers, confirming the sensitivity of the assay and the metabolic activity of S9 preparation. If the validity criteria were met, the test article was considered to be mutagenic if:

1. It produces a two-fold increase in the number of induced revertants compared to the negative control (not-exposed bacteria suspension)
2. Revertant numbers of three or more of the test substance concentrations are significantly higher than the negative control
3. Linear dose-response was observed. This would provide stronger evidence of mutagenic activity
4. The positive responses described above were reproducible

The analysis was performed using the statistical software GraphPad Prism version 8.4.3. In case of results with positive slope in non-threshold model and Dunnett’s test (P value < 0.05), the tests were repeated. A test substance is deemed mutagenic if the effect is confirmed in two replicates.

The micronucleus values obtained by the different test article concentrations were compared pairwise to those from the corresponding negative controls using chi-square analysis for a qualitative analysis. A Cochran–Armitage trend test was performed to check for a dose-response relationship. Criteria to consider a result positive were: 1. A reproducible, dose-dependent increase in MN frequencies should be obtained; 2. The increase should reach statistical significance compared to the study’s negative control and the lab’s historical negative control; 3. The toxicity associated with the statistically significant increase should not exceed 60%. For a reproducible positive response, the quantitative determination of the effective dose necessary to reach the 3-fold increase in micronuclei over background frequencies was performed (ECMN3) on a “per-puff” basis. That is, the higher the ECMN3, the lower the genotoxic potential. For the calculation of the ECMN3, the historical background frequency was subtracted from the single raw data, and a nonlinear regression was applied. According to guidelines, MN values associated with toxicities above 60% were not considered for the qualitative assessment. However, in case of a positive result, MN data associated with toxicities >60% were included for the quantitative assessment to ensure the best curve fit and ECMN3 calculation.

### Clinical study

#### Overall study design

This open-label, randomized, crossover, clinical study was designed to assess nicotine pharmacokinetics and subjective effects among cigarette smokers using the iSENZIA™ HHS. The study received a favorable opinion (equivalent to an ethics approval) from the Office for Research Ethics Committees Northern Ireland (ORECNI) Health and Social Care Research Ethics Committee A (reference number 23-NI-0099) prior to study commencement. The study was performed in accordance with ethical principles set forth in the Declaration of Helsinki and was compliant with the principles and requirements of International Council for Harmonisation (ICH) E6 (R2) guidelines for Good Clinical Practice (GCP), the European Union Clinical Trials Directive, and applicable local regulatory requirements. The study was performed at a single clinical site in Belfast, Northern Ireland (United Kingdom) and was registered in the ClinicalTrials.gov repository (NCT06093659). Twenty-five (25) male and female healthy adult smokers participated in this study. Subjects attended the clinic site on two separate occasions: a screening visit and a 6-day confinement period (Supplementary Figure S2). After completing the study, a follow-up telephone call was conducted for all subjects no longer than 1 week after the end of their confinement period. All subjects provided written informed consent prior to the commencement of any study procedures, including screening assessments.

#### Study subjects

In total, 67 subjects were screened for participation in this study; 42 subjects either did not meet screening criteria (inclusion and exclusion criteria detailed below) or were not needed for study participation (i.e., they were study reserves but were not required to take part in the study). A total of 25 subjects were enrolled, and all subjects completed the study.

Inclusion criteria were that subjects were healthy adults aged 21–65 years inclusive at screening; self-reported smoking at least 10 manufactured combustible (menthol or non-menthol) cigarettes per day (CPD) for at least 12 months prior to screening; had a urine cotinine ≥500 ng/mL at screening; had an exhaled carbon monoxide (eCO) level >10 parts per million (ppm) at screening; if female and of childbearing potential, were using at least one approved form of contraception; if female and of non-childbearing potential, had undergone a sterilization procedure at least 6 months prior to check-in or was postmenopausal with amenorrhea (verified by measuring follicle-stimulating hormone (FSH) levels) for at least 1 year prior to check-in; if a non-vasectomized male, agreed to use a condom with spermicide or abstain from intercourse for duration of the study and extending up to 90 days post-study; if male, agreed not to donate sperm for duration of the study and extending up to 90 days post-study; was willing comply with the requirements of the study, including a willingness to use the study HHP; and provided voluntary consent to participate in this study, which was documented by signing of the signed informed consent form.

Subjects were not allowed to enter the study if any exclusion criteria were met. The main exclusion criteria were a history or presence of clinically significant disease or disorder that, in the opinion of the investigator, would have jeopardized the safety of the subject or impacted the validity of the study results; had a clinically significant abnormal finding on the physical examination, medical history, vital signs, electrocardiogram, or clinical laboratory results; had an acute illness (e.g., upper respiratory tract infection, viral infection) requiring treatment within 14 days prior to check-in; systolic blood pressure (BP) < 90 mmHg or >150 mmHg, diastolic BP < 40 mmHg or >95 mmHg, or heart rate (HR) < 40 bpm or >99 bpm at screening; estimated creatinine clearance (using the Cockcroft Gault equation) < 70 mL/min at screening; used medications known to interact with cytochrome P450 2A6 within 3 months prior to check-in and throughout the study; used inhalers to treat any medical condition within 3 months prior to check-in and throughout the study; used prescription or over-the-counter bronchodilator medication (e.g., inhaled or oral β-agonists) for treatment of any illness within 12 months prior to check-in and throughout the study; was allergic to or could not tolerate flavoring agents used in any of the study products; had used any prescription smoking cessation treatments, including, but not limited to, varenicline (Chantix^®^) or bupropion (Zyban^®^) within 3 months prior to check-in; was planning to quit smoking during the study or within the next 3 months or was postponing a quit attempt in order to participate in the study; or had donated blood or blood products (including plasma), had significant blood loss, or received whole blood or a blood product transfusion within 90 days prior to check-in.

#### Randomization

Subjects who completed the study screening assessments were assigned a unique randomization identification number. Subsequently, each subject, based on the identification number, was assigned to use the study products according to one of five product sequences with five subjects per sequence. Randomization sequences were prepared by Celerion, Inc.

#### Study procedures

This study was a randomized, open-label, crossover, confinement study with 25 male and female cigarette smokers. The study assessed five test products and a cigarette comparator and evaluated nicotine pharmacokinetics, subjective effects, puffing topography, and product safety. Of the five test products, data regarding two HTP products assessed are not described in this article. An overview of the study design is presented in Supplementary Figure S2.

At Visit 1 (screening), which took place within 28 days prior to study procedures on Day −1, subjects underwent numerous assessments to check their eligibility to participate in the study, to review their health status, and to assess their nicotine consumption habits. Screening procedures included a physical examination (including oral cavity and oropharynx), vital signs, electrocardiogram, body mass index (BMI), clinical laboratory tests (hematology, serum chemistry, and urinalysis), serology, urine/saliva drugs of abuse screen, urine/breath alcohol, cotinine screen, eCO, and pregnancy and FSH tests (for females as appropriate). If requested, subjects were offered smoking cessation advice and contact information for a smoking cessation support service.

Visit 2 was a 6-day confinement period. subjects who successfully completed the screening procedures and met all the inclusion criteria and none of the exclusion criteria were eligible to check-in to the clinical site for the confinement period. Subjects checked into the clinic on Day −1 and remained at the clinic until Day 5 for daily study product use, nicotine pharmacokinetics sampling, subjective questionnaire assessments, puffing topography assessments, and safety assessments. On Day −1, following eligibility confirmation, subjects undertook a familiarization session with the study products and the questionnaires. The site’s clinical team explained how the iSENZIA™ HHS stick was to be used. Subjects had the opportunity to see the products/devices and packaging and participated in a product trial in which they took five puffs on a single iSENZIA™ HHS stick of a flavor of their own choosing. An explanation of how the questionnaires were to be administered to the subjects was given. After the familiarization session and completion of check-in procedures, subjects were allowed to smoke their own cigarettes *ad libitum* but abstained from the use of any tobacco- or nicotine-containing products for at least 12 h prior to the start of the controlled product use session on the morning of Day 1. In the morning of Day 1, after pre-use assessments and confirmation of eligibility, the subjects were randomized to one of the five product sequences and then provided a single product of the study product in the sequence to which they had been randomized. On Days 1 through 5, subjects used the assigned study product under controlled conditions (i.e., completely used a single iSENZIA™ HHS stick or smoked a single cigarette), with puffs taken at 30-s intervals and puffs 3 s in duration. Blood samples for nicotine assessment were collected 5 min prior to initiating product use and at 2 min, 4 min, 6 min, 8 min, 10 min, 15 min, 30 min, 45 min, 60 min, 120 min, and 240 min following the start of study product use. Subjective effects questionnaires were administered to the subjects at defined intervals throughout the day to assess the subjects’ urge to smoke, intent to use, and product evaluation. The Urge to Smoke questionnaire was administered 10 min before product use (Time −10), and at 4 min, 8 min, 15 min, 45 min, 60 min, 120 min, and 240 min post-use. Intent to Use and Product Evaluation questionnaires were administered at the 240-min time point. When coinciding with a blood draw, the Urge to Smoke questionnaire was administered approximately 30 s prior to the blood draw. All other questionnaires were completed within approximately 2 min following the final scheduled blood draw at 240 min. Safety was also monitored throughout the day. For all subjects, meals and snacks were provided at the appropriate times during confinement at the clinic site. Each meal and/or snack served at the site was standardized, similar in caloric content and composition, and taken at approximately the same time on each day. When confined at the clinic site, subjects were required to fast from all food and drink except water between meals and snacks.

On Days 1 through 5, following the 4-h pharmacokinetic blood collection period, subjects started a 4-h *ad libitum* product use session (no limits on cigarette or HHS stick consumption) with the same study product as that used during the morning controlled use session. Puffing topography assessments were made at this time but are not reported in this manuscript, and no blood samples were taken in this period for nicotine pharmacokinetic analyses. After completion of the *ad libitum* use session, subjects were allowed to smoke their own cigarettes *ad libitum* until at least 12 h prior to the start of the morning controlled product use session scheduled on the following day. On Day 5, following completion of study assessments, subjects were allowed to smoke their own cigarettes. They left the clinic after completing all final check-out requirements.

A follow-up telephone call (Visit 3) was made by the clinic to contact all subjects using their standard procedures approximately 7 days after the final product use to determine if any adverse events (AEs) had occurred since the last study visit.

#### Nicotine pharmacokinetics

To determine blood plasma nicotine concentrations during and after use of the study products, blood samples (approximately 4 mL) were collected either by direct venipuncture or through an indwelling venous catheter at the time points described above. Blood samples were drawn into dipotassium ethylenediaminetetraacetic acid (K_2_EDTA) vacutainer tubes, and the plasma fraction was separated off by centrifugation and pipetting. Plasma nicotine was analyzed by liquid chromatography-tandem mass spectrometry (LC-MS/MS) at Celerion Bioanalytical Services (Lincoln, Nebraska, United States) using a validated analytical method with appropriate quality controls according to the Food and Drug Administration (FDA) Guidance for Industry (Title 21 Code of Federal Regulations Part 58). Sample processing was completed by a non-tobacco user. The lower limit of quantification of plasma nicotine using the analytical method was 0.2 ng/mL.

#### Subjective effect assessments

The Intent to Use (100-mm visual analog scale (VAS)), Urge to Smoke (VAS), and Product Evaluation Scale (PES; 7-point scale; Hatsukami et al., 2013) questionnaires were completed using a Veeva ePro computerized tablet device (Veeva, Pleasanton, CA, United States). All relevant software and staff training specific to the electronic questionnaires were provided by Celerion, Inc. Any electronic device used met all regulatory requirements, including FDA 21 CFR Part 11. The Urge to Smoke questionnaire collected subjects’ responses to the question “Right now, how much would you like to smoke a cigarette?” with responses ranging from “Not at all” (0) to “A great deal” (100). Responses were collected at Time 0 (pre-product use) and at 4 min, 8 min, 15 min, 45 min, 60 min, 120 min, and 240 min relative to the start of product use on Days 1–5. The Intent to Use questionnaire collected subjects’ responses to the question “If available, how likely are you to buy your assigned study product in the future?” with responses ranging from “Not at all” (0) to “Extremely” (100). Responses were collected at 240 min following the start of study product use on Days 1–5. The 21-item PES questionnaire was completed at 240 min following the start of study product use on each of Days 1–5. PES subscale scores in the domains of satisfaction (items 1, 2, 3, and 12), psychological reward (items 4, 5, 6, 7, and 8), aversion (items 9, 10, 16, and 18), and relief (items 11, 13, 14, 15, and reversed for 20), were generated from the individual items as described previously (Hatsukami et al., 2013). Items 17, 19, and 21 were summarized as individual item scores.

#### Statistical analyses

The safety population comprised all subjects who successfully completed eligibility requirements after checking in to the clinic site and used at least one study product. The outcome population was a subset of the safety population and consisted of subjects who used a study product and had evaluable nicotine pharmacokinetics or subjective effect data. This population was used in the summary and analysis of all data presented in this article.

Due to this being the first study to assess the nicotine pharmacokinetics and subjective effects of the PULZE™ 2.0 heated device used with the iSENZIA™ consumables, no sample size calculations could be performed. However, a sample size of 25 subjects was deemed adequate to meet the study objectives, and this is in line with similar HTP nicotine pharmacokinetic study designs in the literature (e.g., Phillips-Waller et al., 2021a; Maloney et al., 2020; Picavet et al., 2016; McDermott et al., 2023).

#### Demographics

Descriptive statistics are reported for continuous variables (age, weight, height, and BMI), and frequency counts were tabulated for categorical demographic variables (sex, ethnicity, and race). Descriptive statistics are also provided for smoking history variables (cigarettes smoked per day and number of years smoking).

#### Nicotine pharmacokinetics

Unadjusted plasma nicotine concentrations that were below the limit of quantitation (BLQ) were set to one-half of the lower limit of quantitation (LLOQ) for the calculation of descriptive statistics. Individual plasma nicotine concentrations were adjusted for baseline nicotine levels (“baseline-adjusted”), and all pharmacokinetic parameters were calculated based on the adjusted concentrations. Baseline adjustment was performed by subtraction of the pre-existing nicotine concentration from each nicotine concentration obtained after test product administration in that period/day for each subject using the following equation:

$${\text{C}}_{\text{t}}={\text{C}}_{\text{t unadjusted}}-\left[C_{0}\;\cdot \;e^{-\text{Kel}\cdot t1}\right]$$

where C_t_ is the adjusted concentration at time t, C_t unadjusted_ is the observed concentration at time t, C_0_ is the pre-product use concentration (−5 min), Kel = $\frac{\ln\;\left(2\right)}{t½}$ , t½ is 2 h (approximate nicotine half-life), t is the actual sampling time since product administration, and t1 is the actual sampling time since the time of the pre-product use sample. After correction for pre-product use values, negative values were assigned a value of zero, and all other values obtained were reported as is, even if these values were BLQ.

SAS^®^ software (Version 9.4) was used for data presentation and summarization, including descriptive statistics, statistical analyses, summary tables, graphs, and data listings. Descriptive statistics were generated for plasma nicotine concentrations and nicotine pharmacokinetic parameters by study product for all subjects, including sample size (n), arithmetic mean (mean), SD, coefficient of variation (CV%), standard error of the mean (SEM), minimum, median, and maximum at each nominal time point. In addition, geometric mean and geometric CV% are provided for the C_max_ (maximum plasma nicotine concentration) and AUC (area under the plasma nicotine concentration-time curve) parameters. Mean concentration–time profiles are presented on linear scales. Missing data were treated as missing, and no imputation was conducted.

A linear mixed-effects model for analysis of variance (ANOVA) was performed on the natural log-transformed pharmacokinetic parameters C_max_ and AUC_t_ following the morning product use session on each of Days 1–5. The model included sequence, product, and study period as fixed effects and subject-nested-within-sequence as a random effect. Geometric least-squares means (LSMs) and 95% intervals (CIs) are provided for the pharmacokinetic parameters C_max_ and AUC_t_ by study product. Geometric LSM ratios, 95% CIs of the geometric LSM ratios, and p-values are provided for the comparisons of C_max_ and AUC_t_. The comparisons of interest included each of the products compared to each other. These statistical analyses were performed using SAS^®^ PROC MIXED.

A non-parametric analysis (Wilcoxon signed rank test) was performed for the comparisons of T_max_ between each of the study products. The Hodges–Lehman estimate median difference and 95% CI of the difference are presented for each comparison. The CIs were constructed using Walsh averages and the appropriate quantile of the Wilcoxon signed rank test statistic. T_max_ was not log-transformed for these analyses.

### Subjective effects

#### Urge to smoke

The derived parameters E_max_, TE_max_, and AUEC_0–240_ were listed by subject and summarized by product using descriptive statistics, including n, mean, SD, CV%, SEM, minimum, Q1, median, Q3, maximum, and 95% CI. A linear mixed-effects model for ANOVA was used to compare urge to smoke parameters without data transformation; the model includes product sequence, period, and product as fixed effects and subject-nested-within-sequence as a random effect. LSM and 95% CIs are provided for E_max_ and AUEC_0–240_ by study product. LSM differences, 95% CIs of the LSM difference, and p-values are provided for the product comparisons for E_max_ and AUEC_0–240_. The comparisons of interest included each of the products compared to each other. These statistical analyses were performed using SAS^®^ PROC MIXED.

#### Product evaluation scale

Descriptive statistics for the composite (satisfaction, psychological reward, aversion, and relief) and individual (ease of use, comfort using in public, and dependence concerns) PES factor scores (Hatsukami et al., 2013) are summarized by study product.

#### Intent to use

Descriptive statistics for the VAS raw score and bipolar score are summarized for each study product. A frequency count table is presented for the categories of the bipolar scores. Bipolar scores were calculated by subtracting 50 from the original VAS score, then categorizing into three categories: −50 to < 0, 0, and > 0 to 50.

#### Safety assessments

Safety was monitored through physical examination (symptom-driven), vital signs measurements, electrocardiograms, and clinical laboratory tests (serum chemistry, hematology, and urinalysis). Adverse event information was also collected throughout the study. Adverse events (including SAEs) were recorded from the start of the first product used until the end-of-study telephone call. Severity/intensity were graded as mild, moderate, or severe, and AEs were also assessed as unlikely, possibly, or probably related to the study product by the investigator.

## Data Availability

The original contributions presented in the study are included in the article/Supplementary Material, further inquiries can be directed to the corresponding author.
