# Supplementary material for: Assessment of heated herbal products’ tobacco harm reduction potential: pre‐clinical and clinical studies
Source: Front Toxicol. 2025 Aug 11;7:1589480. doi: 10.3389/ftox.2025.1589480 (PMC12375587; doi:10.3389/ftox.2025.1589480)
Supplement: Supplementary file 1 [file Supplementaryfile1.docx]

**Assessment of a Heated Herbal Product's Tobacco Harm Reduction Potential: Pre-Clinical and Clinical Studies**

Alvaro‑Flavio Marinas-Lacasta^1^, Ian M. Fearon^2^, Matthew Stevenson ^§1^, Tasnim Abusalem^1^, Fiona Chapman^1^, Edgar Trelles-Sticken^3^, Roman Wieczorek^3^, Sarah Jean Pour^3^, Ole Dethloff^3^, Ourania Komini^1^, Mike Brown^1^, Liam Simms^1^, and Thomas Nahde^3^

^1^Imperial Brands PLC, 121 Winterstoke Road, Bristol, BS3 2LL, UK; ^2^whatIF? Consulting Ltd, The Crispin, Burr Street, Harwell, OX11 0DT, U.K.; ^3^Imperial Brands Reemtsma, Behringstrasse 122a, 22763 Hamburg, Germany

^§^**Corresponding author**

Matthew Stevenson

Imperial Brands PLC

121 Winterstoke Road

BS3 2LL

Bristol, UK

Email: matthew.stevenson@impbrands.com

**Supplementary Figure 1. iSENZIA^TM^ Heated Herbal System Configuration.**

| **A** | **B** |
| --- | --- |
| **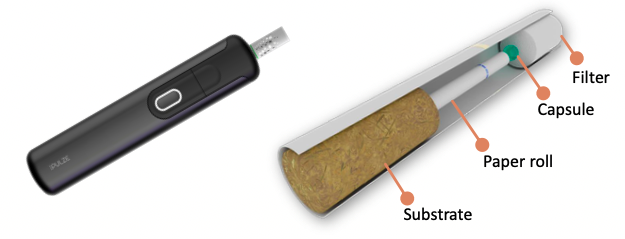** | |

Images are (**A**) the PULZE 2.0 heating device which is electrically powered and designed to heat the herbal tea‑based substrate of the iSENZIA^TM^ HHS sticks to generate a nicotine‑containing aerosol; and (**B**) the iSENZIA^TM^ HHS stick, which contains a filter, a flavour capsule (“crushball”) which contains a different flavour liquid depending on the particular stick variant, capsule positioning‑ paper rolls, and a substrate which is tea‑based and contains other components including cellulose, nicotine, a binder, and a variety of flavour ingredients. Abbreviation: HHS, heated herbal system.

**Supplementary Figure 2. Clinical Study Design Overview.**

| **VISIT 1** |  | **VISIT 2** | | | | | | | | | |  | **VISIT 3** |
| --- | --- | --- | --- | --- | --- | --- | --- | --- | --- | --- | --- | --- | --- |
|  |  |  |  | | |  | |  | |  | |  |  |
| Day ‑28 to  Day ‑1 |  | Day ‑1 | Day 1 | Day 2 | | | Day 3 | | Day 4 | | Day 5 |  | Day 12 |
|  |  |  |  | |  | | |  | |  | |  |  |
| SCREENING |  | CLINICAL CONFINEMENT | | | | | | | | | |  | END OF STUDY |
|  |  |  | | | | | | | | | |  |  |
|  |  | RANDOMISATION | On each day, controlled use of a single iD (HTP) or iSENZIA^TM^ HHS stick, or smoking of a single cigarette  3‑second puffs taken at 30‑second intervals | | | | | | | | |  |  |
|  |  |  |  | |  | | |  | |  | |  |  |
|  |  |  |  | |  | | |  | |  | |  |  |
| Health Status  Eligibility |  | Days 1, 2, 3 and 4  Plasma nicotine sampling for nicotine pharmacokinetic assessment  Subjective effects questionnaire administration  Safety assessments | | | | | | | | | |  | Telephone call safety follow‑up |

Abbreviations: HTP, heated tobacco product; HHS, heated herbal system.

Supplementary Table 1. Levels of WHO TobReg 9 Analytes, ACM and Nicotine in iSENZIA^TM^ HHS Whole Aerosol Compared with 1R6F Reference Cigarette Smoke.

|  |  |  | **iSENZIA^TM^ HHS *Forest Berry*** | | **iSENZIA^TM^ HHS *Summer Watermelon*** | | **1R6F Reference Cigarette** | | **% Reduction (Per Puff)** | |
| --- | --- | --- | --- | --- | --- | --- | --- | --- | --- | --- |
|  | **Analyte** | **Units** | **Per stick** | **Per puff** | **Per stick** | **Per puff** | **Per stick** | **Per puff** | **iSENZIA^TM^ HHS *Forest Berry*** | **iSENZIA^TM^ HHS *Summer Watermelon*** |
| **WHO TobReg 9 analytes** | **CO** | mg | 0.38 | 0.038 | 0.38 | 0.038 | 28.77 | 3.16 | 98.79 | 98.79 |
|  | **NNN** | ng | <LOQ | <LOQ | <LOQ | <LOQ | 219.73 | 24.97 | 98.58 | 98.58 |
|  | **NNK** | ng | <LOQ | <LOQ | <LOQ | <LOQ | 177.73 | 20.20 | 98.25 | 98.25 |
|  | **Benzo[a]pyrene** | ng | <LOQ | <LOQ | <LOQ | <LOQ | 13.81 | 1.61 | 88.99 | 88.99 |
|  | **Formaldehyde** | µg | <LOQ | <LOQ | <LOQ | <LOQ | 107.80 | 10.44 | 98.93 | 98.93 |
|  | **1,3‑butadiene** | µg | <LOQ | <LOQ | <LOQ | <LOQ | 103.89 | 12.50 | 99.66 | 99.66 |
|  | **Acetaldehyde** | µg | 50.9 | 5.09 | 51.0 | 5.10 | 1132.8 | 136.32 | 96.27 | 96.26 |
|  | **Acrolein** | µg | 2.22 | 0.22 | 2.29 | 0.23 | 164.74 | 19.82 | 98.88 | 98.84 |
|  | **Benzene** | µg | <LOQ | <LOQ | <LOQ | <LOQ | 76.99 | 9.26 | 99.79 | 99.79 |
| **ACM + nicotine** | **ACM/TPM** | mg | 34.5 | 3.45 | 34.47 | 3.447 | 38.37 | 4.22 | 18.25 | 18.25 |
|  | **Nicotine** | mg | 0.72 | 0.07 | 0.69 | 0.069 | 1.88 | 0.21 | 64.99 | 66.76 |

Analyte yields are presented as mean values from 3 replicates on either a per stick or a per puff basis, as indicated. Percentage reductions for those analytes which were <LOQ for the iSENZIA^TM^ HHS were calculated by converting the LOQ to a per-puff amount and then dividing this value by the square root of 2. 1R6F reference cigarettes were smoked on a rotary smoking machine according to the ISO 20778:2018 smoking regimen (55ml puff volume, 2s puff duration, 30s puff interval, bell-shaped puff profile, filter vents blocked). The iSENZIA*™* HHS was used on a linear smoking machine according to a modified version of ISO 20778:2018 (55ml puff volume, 2s puff duration, 30s puff interval, bell-shaped puff profile, with no filter vent blocking). Abbreviations: WHO TobReg 9, World Health Organization Study Group on Tobacco Product Regulation proposal of toxicants mandated for lowering in cigarette smoke ^[73]^; HHS, heated herbal system; ACM, aerosol collected mass; TPM, total particulate matter; NNN, *N*‑nitrosonornicotine; NNK, 4‑(methylnitrosamino)‑1‑(3‑pyridyl)‑1‑butanone; LOQ, limit of quantitation.

Supplementary Table 2. Summary of Clinical Study Subject Demographics.

|  |  | **Randomised Product Sequence^1^** | | | | |  |
| --- | --- | --- | --- | --- | --- | --- | --- |
|  |  | **ABECD** | **BCADE** | **CDBEA** | **DECAB** | **EADBC** | **Overall** |
| **Trait** | n | 5 | 5 | 5 | 5 | 5 | 25 |
| Sex | Female | 2 (40%) | 2 (40%) | 2 (40%) | 2 (40%) | 2 (40%) | 10 (40%) |
|  | Male | 3 (60%) | 3 (60%) | 3 (60%) | 3 (60%) | 3 (60%) | 15 (60%) |
| Race | White | 5 (100%) | 5 (100%) | 5 (100%) | 5 (100%) | 5 (100%) | 25 (100%) |
| Ethnicity | Not Hispanic or Latino | 5 (100%) | 5 (100%) | 5 (100%) | 5 (100%) | 5 (100%) | 25 (100%) |
| Age (years) | Mean | 46.4 | 39.4 | 43.2 | 36.4 | 37.8 | 40.6 |
|  | SD | 9.91 | 8.62 | 8.70 | 12.58 | 6.06 | 9.38 |
|  | Minimum | 35 | 28 | 34 | 23 | 31 | 23 |
|  | Median | 47.0 | 37.0 | 44.0 | 40.0 | 40.0 | 40.0 |
|  | Maximum | 58 | 51 | 54 | 52 | 45 | 58 |
| Weight (kg) | Mean | 73.3 | 75.9 | 75.3 | 66.4 | 77.1 | 73.6 |
|  | SD | 11.47 | 2.90 | 9.42 | 9.12 | 12.01 | 9.54 |
|  | Minim | 55.1 | 71.4 | 61.8 | 56.3 | 56.3 | 55.1 |
|  | Median | 75.4 | 77.1 | 75.4 | 62.7 | 83.8 | 75.4 |
|  | Maximum | 84.1 | 78.6 | 87.9 | 79.5 | 84.5 | 87.9 |
| Height (cm) | Mean | 169.4 | 172.8 | 171.8 | 170.6 | 171.4 | 171.2 |
|  | SD | 7.20 | 2.77 | 6.72 | 6.99 | 4.98 | 5.58 |
|  | Minimum | 160 | 169 | 161 | 163 | 166 | 160 |
|  | Median | 171.0 | 174.0 | 174.0 | 170.0 | 172.0 | 171.0 |
|  | Maximum | 176 | 176 | 178 | 182 | 179 | 182 |
| BMI (kg/m²) | Mean | 25.5 | 25.4 | 25.6 | 22.8 | 26.2 | 25.1 |
|  | SD | 3.11 | 1.57 | 3.85 | 2.72 | 3.34 | 3.00 |
|  | Minimum | 20.5 | 23.6 | 19.5 | 19.9 | 20.4 | 19.5 |
|  | Median | 27.2 | 25.6 | 27.1 | 21.7 | 27.3 | 26.2 |
|  | Maximum | 27.8 | 27.0 | 29.0 | 26.9 | 28.6 | 29.0 |
| Cigarettes Smoked per Day | 10‑14 | 1 (20%) | 1 (20%) | 1 (20%) | 1 (20%) | 2 (40%) | 6 (24%) |
|  | 15‑19 | 1 (20%) | 3 (60%) | 3 (60%) | 3 (60%) | 1 (20%) | 11 (44%) |
|  | 20‑29 | 3 (60%) | 1 (20%) | 1 (20%) | 1 (20%) | 2 (40%) | 8 (32%) |
| Number of Years Smoking Cigarettes | Mean | 27.0 | 22.4 | 25.6 | 15.4 | 15.2 | 21.1 |
|  | SD | 8.60 | 9.07 | 9.50 | 7.92 | 9.52 | 9.62 |
|  | Minimum | 18 | 12 | 16 | 6 | 6 | 6 |
|  | Median | 29.0 | 19.0 | 26.0 | 15.0 | 14.0 | 19.0 |
|  | Maximum | 36 | 35 | 35 | 26 | 25 | 36 |

^1^Individual product codes were (A) iD^TM^ Balanced Blue (HTP) sticks, (B) iD^TM^ Rich Bronze (HTP) sticks, (C) iSENZIA^TM^ HHS *Forest Berry*, (D) iSENZIA^TM^ HHS *Summer Watermelon*, and (E) subjects’ usual brand cigarettes. Abbreviations: BMI, body mass index; SD, standard deviation; HHS, heated herbal system; HTP, heated tobacco product.

**Supplementary Table 3. Summary of Product Use in the Controlled Puffing Sessions of the Clinical Study.**

| **Variable** | **iSENZIA^TM^ HHS *Forest Berry*** | **iSENZIA^TM^ HHS *Summer Watermelon*** | **Usual brand cigarettes** |
| --- | --- | --- | --- |
| n | 25 | 25 | 25 |
| Mean | 9.3 | 9.3 | 10.3 |
| SD | 1.28 | 1.28 | 1.70 |
| CV(%) | 13.8 | 13.7 | 16.5 |
| SEM | 0.26 | 0.26 | 0.34 |
| Minimum | 7 | 7 | 7 |
| Median | 10.0 | 9.0 | 10.0 |
| Maximum | 11 | 12 | 14 |
| 95% CI | 8.8, 9.8 | 8.8, 9.8 | 9.6, 11.0 |

Abbreviations: n, number of observations; SD, standard deviation; CV(%), coefficient of variation; CI, confidence intervals; HHS, heated herbal system.

**Supplementary Table 4. Summary of Statistical Comparisons of the Baseline‑Adjusted Plasma Nicotine Pharmacokinetic Parameters C_max_ and AUC_t_ in the Outcomes Population of the Clinical Study.**

|  |  | **Geometric LS Mean** | |  |  |  |
| --- | --- | --- | --- | --- | --- | --- |
| **Product Comparison** | **Parameter** | **Test (n)** | **Reference (n)** | **% Geometric LS Mean Ratio (Test/Reference)** | **95% CI** | **p value** |
| **iSENZIA^TM^ HHS *Forest Berry* versus usual brand cigarettes** | C_max_ | 5.296 (25) | 16.44 (25) | 32.20 | 25.80, 40.20 | <0.0001 |
|  | AUC_t_ | 343.0 (25) | 1078 (25) | 31.82 | 26.99, 37.51 | <0.0001 |
| **iSENZIA^TM^ HHS *Summer Watermelon* versus usual brand cigarettes** | C_max_ | 6.164 (25) | 16.44 (25) | 37.48 | 30.03, 46.79 | <0.0001 |
|  | AUC_t_ | 357.3 (25) | 1078 (25) | 33.15 | 28.12, 39.09 | <0.0001 |
| **iSENZIA^TM^ HHS *Forest Berry* versus iSENZIA^TM^ HHS *Summer Watermelon*** | C_max_ | 5.296 (25) | 6.164 (25) | 85.91 | 68.82, 107.25 | 0.1773 |
|  | AUC_t_ | 343.0 (25) | 357.3 (25) | 95.98 | 81.40, 113.16 | 0.6217 |

The mixed model included sequence, product, and study period as fixed effects and subject‑nested‑within‑sequence as a random effect. Mixed model with a default (variance component) covariance structure was used. Parameters were ln‑transformed prior to analysis. Geometric LS means were calculated by exponentiating the LS Means from the ANOVA. Abbreviations: HHS, heated herbal system; C_max_, maximum plasma nicotine concentration; AUC_t_, area under the plasma nicotine concentration‑time curve from zero to the time of the last measurable non‑zero concentration; LS, least‑squares; CI, confidence interval; n, number of observations.

Supplementary Table 5. Summary of Nonparametric Statistical Comparison of the Baseline‑Adjusted Plasma Nicotine Pharmacokinetic Parameter T_max_ in the Outcomes Population of the Clinical Study.

|  | **Difference (Test – Reference)** | |  |
| --- | --- | --- | --- |
| **Comparison** | **Median** | **95% Confidence Interval** | **p‑value** |
| **iSENZIA^TM^ HHS *Forest Berry* versus usual brand cigarettes** | ‑1.40 | ‑1.874, 0.083 | 0.1076 |
| **iSENZIA^TM^ HHS *Summer Watermelon* versus usual brand cigarettes** | ‑0.333 | ‑1.917, 0.059 | 0.0902 |
| **iSENZIA^TM^ HHS *Forest Berry* vs iSENZIA^TM^ HHS *Summer Watermelon*** | ‑0.0160 | ‑0.959, 1.000 | 0.9792 |

The 95% confidence interval was constructed using Walsh Averages and appropriate quantile of the Wilcoxon Signed Rank test statistic. Abbreviations: HHS, heated herbal system; T_max_, time of the maximum plasma nicotine concentration.

**Supplementary Table 6. Summary of Statistical Comparisons of the Urge to Smoke Parameters E_max_ and AUEC_0‑240_ in the Outcomes Population of the Clinical Study.**

|  |  | **LS Means** | |  |  |  |
| --- | --- | --- | --- | --- | --- | --- |
| **Product Comparison** | **Parameter** | **Test (n)** | **Reference (n)** | **LS Means Difference (Test ‑ Reference)** | **95% CI** | **p value** |
| **iSENZIA^TM^ HHS *Forest Berry* versus usual brand cigarettes** | E_max_ | 42.96 (25) | 69.20 (25) | ‑26.24 | ‑38.64, 13.84 | <.0001 |
|  | AUEC_0‑240_ | 3369 (25) | 6083 (25) | ‑2714 | ‑4802.96, 624.35 | 0.0115 |
| **iSENZIA^TM^ HHS *Summer Watermelon* versus usual brand cigarettes** | E_max_ | 44.28 (25) | 69.20 (25) | ‑24.92 | ‑37.32, 12.52 | 0.0001 |
|  | AUEC_0‑240_ | 3228 (25) | 6083 (25) | ‑2854 | ‑4943.55, 764.94 | 0.0080 |
| **iSENZIA^TM^ HHS *Forest Berry* versus iSENZIA^TM^ HHS *Summer Watermelon*** | E_max_ | 42.96 (25) | 44.28 (25) | ‑1.320 | ‑13.72, 11.08 | 0.8331 |
|  | AUEC_0‑240_ | 3369 (25) | 3228 (25) | 140.6 | ‑1948.72, 2229.90 | 0.8940 |

The mixed model includes product sequence, period, and product as fixed effects and subject nested within product sequence as a random effect. Mixed model with a default (variance component) covariance structure was used. Least‑squares means (LS means) were calculated from the ANOVA. Abbreviations: HHS, heated herbal system; E_max_, maximum change in urge to smoke; AUEC_0‑240_, area under the effect‑time curve from zero to the 240 minutes; LS, least‑squares; CI, confidence interval; n, number of observations.
